# Fluorescent Silicon Nanorods-Based Nanotheranostic Agents for Multimodal Imaging-Guided Photothermal Therapy

**DOI:** 10.1007/s40820-019-0306-9

**Published:** 2019-09-09

**Authors:** Mingyue Cui, Sangmo Liu, Bin Song, Daoxia Guo, Jinhua Wang, Guyue Hu, Yuanyuan Su, Yao He

**Affiliations:** Laboratory of Nanoscale Biochemical Analysis Institute of Functional Nano & Soft Materials (FUNSOM), and Jiangsu Key Laboratory for Carbon-Based Functional Materials & Devices, Soochow University, 199 Ren’ai Road, Suzhou, 215123 Jiangsu People’s Republic of China

**Keywords:** Gold nanoparticle, Fluorescent silicon nanorods, Nanotheranostic, Multimodal imaging, Photothermal therapy, Tumor target

## Abstract

The utilization of diagnosis to guide/aid therapy procedures has shown great prospects in the era of personalized medicine along with the recognition of tumor heterogeneity and complexity. Herein, a kind of multifunctional silicon-based nanostructure, i.e., gold nanoparticles-decorated fluorescent silicon nanorods (Au@SiNRs), is fabricated and exploited for tumor-targeted multimodal imaging-guided photothermal therapy. In particular, the prepared Au@SiNRs feature high photothermal conversion efficiency (~ 43.9%) and strong photothermal stability (photothermal performance stays constant after five-cycle NIR laser irradiation), making them high-performance agents for simultaneously photoacoustic and infrared thermal imaging. The Au@SiNRs are readily modified with targeting peptide ligands, enabling an enhanced tumor accumulation with a high value of ~ 8.74% ID g^−1^. Taking advantages of these unique merits, the Au@SiNRs are superbly suitable for specifically ablating tumors in vivo without appreciable toxicity under the guidance of multimodal imaging. Typically, all the mice treated with the Au@SiNRs remain alive, and no distinct tumor recurrence is observed during 60-day investigation.

**Electronic supplementary material:**

The online version of this article (10.1007/s40820-019-0306-9) contains supplementary material, which is available to authorized users.

## Highlights

- A kind of multifunctional silicon-based theranostic agent is fabricated and exploited for imaging-guided tumor-targeted photothermal therapy.
- The obtained gold nanoparticles-decorated fluorescent silicon nanorods featuring high photothermal conversion performance and good photothermal stability enable a total ablation of tumors and prolong the survival time of mice.

**Electronic supplementary material:**

The online version of this article (10.1007/s40820-019-0306-9) contains supplementary material, which is available to authorized users.

## Graphical abstract

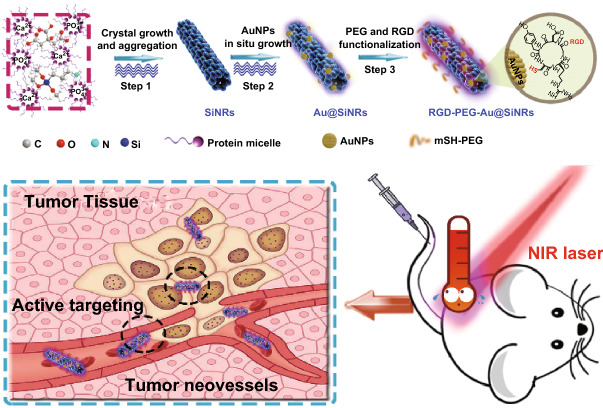

## Introduction

Along with tremendous advances in cancer nanomedicine, more challenges such as the complexity and heterogeneity of tumors are gradually realized [1, 2]. Using diagnosis to guide/aid therapy procedures would show great prospects in the era of personalized medicine [3, 4]. To enhance the diagnosis accuracy, different imaging modalities are expected to be integrated together [5–9]. However, besides tedious manipulations, the rational integration of two or more imaging modalities into one therapy platform normally suffers from low yield and instability of products. As a consequence, great efforts are currently needed for the development of novel all-in-one multimodal imaging-based nanoplatform.

On the other hand, it is of particular interest to develop functional silicon nanostructures for biological and biomedical applications over the years, since silicon nanostructures possess several intrinsic advantages like excellent optical/electronic properties, favorable biocompatibility, and good biodegradability [10–17]. Typically, zero-dimensional fluorescent silicon nanoparticles with robust photostability and negligible toxicity have been extensively explored for real-time and long-term bioimaging [18–23]. One-dimensional silicon nanowires (SiNWs) have been developed as electrochemical and optical biosensors for detecting various biological targets in highly sensitive and specific manner [24–27]. Of note, great efforts have recently been devoted for the exploitation of new-type one-dimensional fluorescent silicon nanostructures, i.e., silicon nanorods (SiNRs), which have drawn intensive attentions in optoelectronics and photovoltaics because of their unique optical properties (e.g., longer Auger lifetimes and higher carrier multiplication quantum yield than zero-dimensional silicon nanoparticles) [28–31]. Lately, a kind of SiNRs-based ratiometric biosensor, featuring strong photostability, good biocompatibility, and broad detection range, was developed for investigating the intracellular pH fluctuation in a long-term and real-time manner [32]. It is worthwhile to point out that recent studies have revealed that the elongated nanostructures like nanorods could exhibit special bio-behaviors such as rapid tumor penetration and enhanced tumor accumulation [33–40]. Therefore, unique optical and special bio-behavioral properties make fluorescent SiNRs promising nanotheranostic agent for cancer diagnosis and therapy, which nevertheless remains vacant up to present.

We herein present the first example of silicon-based theranostic agent for multimodal imaging-guided tumor-targeted photothermal therapy (PTT). The agent is made of gold nanoparticles-decorated fluorescent silicon nanorods (Au@SiNRs), which are prepared via in situ growth AuNPs on microwave-synthesized SiNRs. Remarkably, the obtained Au@SiNRs feature high photothermal conversion performance (photothermal conversion efficiency: ~ 43.9%) and robust photothermal stability (preserving the same temperature elevation curve and morphology after five cycles of NIR laser irradiation), thus suitable for photoacoustic (PA) and infrared thermal imaging. After the surface modification with poly(ethylene glycol) (PEG) and targeting peptide ligands [one cyclic peptide containing the specific sequence of arginine-glycine-aspartic acid (named as c(RGDyC))], the as-fabricated active targeting RGD-PEG-Au@SiNRs have a significantly enhanced tumor accumulation (~ 8.74% ID g^−1^). Moreover, one-time irradiation with an 808-nm NIR laser at a low power density (0.8 W cm^−2^) induces the total ablation of tumors and drastically prolonged survival time of mice.

## Experimental

### Preparation of SiNRs and Au@SiNRs

The microwave system NOVA 2S used to synthesize nanostructures was purchased from Preekem of Shanghai, China. SiNRs were readily achieved through microwave synthesis according to our previous protocol [30]. In detail, precursor solution was prepared through adding 1 mL (3-Aminopropyl)trimethoxysilane (APTES, 97%, bought from Sigma-Aldrich) to 8 mL N_2_-saturated trisodium citrate (99.0%, bought from Sinopharm Chemical Reagent Co., Ltd, China) aqueous solution (0.075 g). After that, 30 mg of milk was introduced into the aqueous solution, followed by 15-min stirring. After transferring to the exclusive vitreous vessel, the mixture was irradiated in the microwave system for 2 h at 150 °C. The impurities of free reagents were excluded from the as-prepared SiNRs by dialysis (1 kDa) and centrifugation (8000 rpm, 5 min/time, 3 times). Gold nanoparticles (AuNPs) were grown on the surface of SiNRs in situ by reducing chloroauric acid (HAuCl_4_, bought from Nanjing Chemical Reagent Co., Ltd, China) with –NH_2_ groups on SiNRs [41]. Briefly, 100 μL of HAuCl_4_ aqueous solutions with different concentrations (5, 10, 15, or 20 mM) was added into 10 mL SiNRs (5.5 mg mL^−1^) suspension. After 40-min stirring and transferring into exclusive vitreous vessel, the Au@SiNRs were prepared through microwave irradiation (MWI) (1.5 h, 120 °C), and then collected by centrifugation (14,800 rpm, 5 min) when the temperature naturally cooled to lower than 30 °C. Before the following process, the sample was washed with deionized water for three times at least. Afterward, the content of gold element in Au@SiNRs was measured by inductively coupled plasma optical emission spectroscopy (ICP-OES). The contention of gold was measured to be 0.56 μg mL^−1^ when the concentration of as-prepared Au@SiNR was 300 μg mL^−1^. Based on this result, the yield of Au@SiNPs was calculated to be ~ 51% on the amount of gold element.

### PEGylation of Au@SiNRs

In order to enhance the biocompatibility and water dispersibility of Au@SiNRs, 20 mg of Au@SiNRs powders was dispersed in 20 mL of deionized water and ultrasonicated for 30 min. Then, 20 mL methoxy-poly (ethylene glycol)-thiol (PEG-SH, molecular weight = 5 KD, bought from Kaizheng Biotech., Beijing, China) (20 mg mL^−1^) aqueous solution was added into the Au@SiNRs suspension, and the mixture was stirred for 24 h under dark condition. The prepared PEGylated Au@SiNRs were washed three times with deionized water by centrifugation at 13,000 rpm for 5 min.

### Preparation of RGD-PEG-Au@SiNRs

The peptide c(RGDyC) (bought from Apeptide (Shanghai) Co., Ltd), which was known as a tumor-specific targeting ligand for selectively binding α_5_β_1_ and α_v_β_3_ integrins, was used to modified Au@SiNRs [42]. The Au@SiNRs was conjugated with c(RGDyC) molecules containing the -SH groups via Au–S bond through the established protocols [43, 44]. Briefly, the mixture of 50 μL peptide c(RGDyC) (50 mM, pH = 6.8) and 100 μL PEGylated Au@SiNRs (~ 10 mg mL^−1^, pH = 7.2) was gently shook in dark at 25 °C for 12 h to produce the c(RGDyC)-modified Au@SiNRs (RGD-PEG-Au@SiNRs). To purify the as-prepared RGD-PEG-Au@SiNRs, 3 kDa Nanosep centrifugal devices were used via centrifugation for 15 min at the speed of 6000 rpm. The sample was stored at 4 °C in the dark after dispersion in phosphate buffer saline (PBS).

### Physicochemical Characterization

Transmission electron microscopy (TEM) overview images were taken at 200 kV through Philips CM 200 electron microscope and analyzed through the software of ImageJ. The atomic and weight fraction of elements existing in the as-prepared materials was taken via energy-dispersive X-ray (EDX) spectroscopy. High-resolution X-ray photoelectron spectroscopy (XPS) spectra were obtained on a Kratos AXIS UltraDLD ultrahigh vacuum (UHV) surface analysis system. Powder UV–vis-NIR absorption spectra were collected with a PerkinElmer Lambda 750 UV–vis-NIR spectrophotometer. Photoluminescence (PL) measurements were performed with a Horiba Jobin–Yvon Fluoromax-4 spectrofluorometer. Fourier transform infrared spectrometer (FTIR) spectra were conducted with a Bruker Hyperion FTIR spectrometer and cumulated scans at a resolution of 4 cm^−1^. The dynamic light scattering (DLS) and zeta potential of materials were detected using Malvern ZEN3690.

### Photoacoustic Signals Detection

To investigate the PA signal-generating ability of Au@SiNRs, gradient concentrations of Au@SiNRs (0, 75, 150, 300, and 400 μg mL^−1^) were added into Eppendorf tubes (200 μL). Then, tubes were embedded into ultrasound gel and subjected to laser illumination in a PA imaging system (Visualsonic Vevo 2100 LAZER system). The wavelength of laser was set at 710 nm.

### Calculation of the Photothermal Conversion Efficiency (η)

To evaluate the value of η, the temperature change of 1.0 mL Au@SiNRs and RGD-PEG-Au@SiNRs aqueous dispersion (0, 75, 150, 300, or 400 μg mL^−1^) was recorded as a function of time under continuous irradiation by the 808 nm laser (1.2 W cm^−2^) for 10 min. Then, the laser was turned off for cooling down to the initial temperature. The result was processed according to the established method [45–47]. The value of η was calculated from Eq. 1, where *T*_max_ is the maximum temperature induced by nanorods, *T*_max, water_ is the maximum temperature induced by pure water, *I* is the incident laser power, and *A*_808_ is the absorption of nanorods dispersed in water at 808 nm. The maximum temperature of water, Au@SiNRs (300 μg mL^−1^) and RGD-PEG-Au@SiNRs (300 μg mL^−1^) was 30.3 °C, 64.7 °C, and 55.9 °C respectively; laser power was set as 1.2 W, and the value of *A*_808_ for Au@SiNRs and RGD-PEG-Au@SiNRs was measured to be 0.678 and 0.528, respectively. The value of *hS* can be obtained through Eqs. 2 and 3, where *mi* and *C*_p, i_ are the mass (1.0 g) and heat capacity (4.2 J g^−1^) of water, *τs* is the sample system time constant, determined by the slope of the linear fit of experimental data plotted according Eq. 3, as shown in Fig. S7 (345.65 and 384.12 s for Au@SiNRs and RGD-PEG-Au@SiNRs). As a result, the photothermal conversion efficiency of Au@SiNRs and RGD-PEG-Au@SiNRs is calculated to be 43.9% and 36.1%.

1 $$\eta = \frac{{hS \left( {T_{\hbox{max} } - T_{{\hbox{max} ,{\text{water}}}} } \right)}}{{I\left( {1 - 10^{{ - A_{808} }} } \right)}}$$

2 $$hS = \frac{{\mathop \sum \nolimits_{i} m_{i} C_{p,i} }}{{\tau_{s} }}$$

3 $$\tau_{s} = - \ln \frac{{T\left( t \right) - T_{\text{sur}} }}{{T_{ \hbox{max} } - T_{\text{sur}} }}$$

### Cytotoxicity Assessment

For evaluating the cytotoxicity of Au@SiNRs, CT-26 cells were plated in 96-well plates at a density of 104 cells per well and incubated for 12 h. Then, old medium was replaced with fresh medium with gradient concentrations of PEG-Au@SiNRs (100 μL per well, 0 ~ 320 μg mL^−1^). Cells were treated with the materials for 24 or 48 h at 37 °C, respectively, and the cytotoxicity was evaluated through MTT (3-(4,5-dimethylthiazol-2-yl)-2,5-diphenyltetrazolium bromide) assay. The efficacy of in vitro photothermal therapy was also investigated in this way. CT-26 cells were plated in a 24-well plate with a density of 105 cells per well. After 12-h incubation, the old medium was replaced by medium containing PEG-Au@SiNRs or RGD-PEG-Au@SiNRs with different concentrations (1 mL per well, 0 ~ 320 μg mL^−1^). After 4-h treatment, the medium was removed, and cells were washed with PBS (PH 7.4) for 2 times. Then, 200 μL PBS was added in each well. Cells were irradiated by an 808 nm laser (0.8 W cm^−2^) for 5 min. Finally, the cell viability was accessed by MTT assay.

### Live/Dead Cell Staining

CT-26 cells were placed in the 24-well plate at a density of 10^5^ cells/well and incubated for another 12 h. Then, 1 mL of fresh medium (blank control group) and medium containing 200 μg mL^−1^ PEG-Au@SiNRs or RGD-PEG-Au@SiNRs (experiment groups) was added. The treated cells were divided into two groups: W group (with NIR) and W/O (without NIR) group. For the W group, after the incubation for 4 h, cells were washed with PBS three times and treated with NIR (808 nm, 0.8 W cm^−2^, 5 min). For the W/O group, cells were washed with PBS for three times without NIR treatment. Finally, cells were stained by live/dead dyes (calcein-AM and propidium iodide dyes) according to the product protocol. The stained cells were recorded with the laser-scanning confocal microscope (LSCM, Leica, TCS-SP5) at 20 × objective.

### Fluorescent Cell Labeling

CT-26 (integrin α_5_β_1_^+^ positive) and 4T1 (integrin α_5_β_1_^−^ negative) cells were plated onto 24-well plate with a density of 105 cells per well, and then incubated for 24 h (37 °C, 5% CO_2_). To label integrin α_5_β_1_, the cells were cultured with 100 μg mL^−1^ PEG-Au@SiNRs, RGD-PEG-Au@SiNRs, or RGD-PEG-Au@SiNRs in the presence of 1 μM of c(RGDyC) (blocking) in a binding buffer (pH 7.4) (37 °C, 5% CO_2_) for 1 h. (For the blocking group, cells were pre-treated with 1 μM of c(RGDyC) for 30 min.) After incubation, cells were washed by PBS (pH 7.4) three times. The labeled cells were mounted on slides in fluoromount (Sigma, F4680) with coverslips. Cell images were captured through LSCM. Imaging was carried out under 40% power of argon laser (λ_ex_ = 405 nm), and the emissions ranging from 425 to 550 nm were recorded.

### Tumor Xenograft

BALB/c nude mice (female, 6–7-week old) were selected to establish the xenograft mice models. 1.5 × 10^6^ CT-26 cells in 125 μL of PBS were subcutaneously inoculated in each mouse at the back. The BALB/c nude mice and BALB/c mice were cared and used under protocols approved by Soochow University Laboratory Animal Center.

### ICP-OES Analysis for Au Element Quantification

For bio-distribution analysis, the absolute Au contents were measured by ICP-OES (Thermo Scientific iCAP6300). When the tumors reached a uniform size of around 80 mm^3^, two groups (*n* = 3 in each group) of the mice were intravenously (i.v.) injected with 200 µL of PEG-Au@SiNRs and RGD-PEG-Au@SiNRs (20 mg kg^−1^) suspensions, respectively. Major organs (heart, liver, spleen, lung, kidney, and tumor) from mice were collected 24 h after i.v. injection. All those organs were weighed and solubilized by aqua regia for ICP-OES measurement.

### In Vivo Photoacoustic Imaging

The PA signal generated by Au@SiNRs was applied in the first imaging strategy. Before PA imaging, the CT-26 tumor-bearing nude BALB/c nude mice were i.v. injected with PEG-Au@SiNRs or RGD-PEG-Au@SiNRs at a dose of 10 mg kg^−1^. The PA signal of the tumor region was detected at different time points (0, 12, and 24 h) through a PA imaging system (Visualsonic Vevo 2100 LAZER system) with an excitation wavelength at 710 nm.

### In Vivo PTT Treatment

CT-26 tumor-bearing mice were randomly divided into ten groups (*n* = 5 per group) when the tumors reached a uniform size of around 80 mm^3^: (a) PBS only; (b) SiNRs only; (c) AuNPs only; (d) PEG-Au@SiNRs only; (e) RGD-PEG-Au@SiNRs only; (f) PBS + NIR; (g) AuNPs + NIR; (h) SiNRs + NIR; (i) PEG-Au@SiNRs + NIR; (j) RGD-PEG-Au@SiNRs + NIR; They were i.v. administered with 200 μL of PBS, SiNRs, AuNPs, PEG-Au@SiNRs, or RGD-PEG-Au@SiNRs (equivalent to 20 mg SiNRs or 0.62 mg Au NPs per kg mouse) suspension, respectively. After 24 h, for groups (f)–(j), the tumor region of the mice was irradiated with an 808 nm NIR laser (Hi-Tech Optoelectronics Co., Ltd. Beijing, China) at a power density of 0.8 W cm^−2^ for 10 min with time interval 30 s. During treatment, the surface temperature of tumors was monitored by an IR thermal camera (Fortric 225). After treatment, the tumor volumes of all the groups were monitored every 2 days with a digital caliper, and the tumor volume was calculated by the following formula:

4 $$\left( {V_{\text{tumor}} = \frac{{{\text{Width}}^{2} \times {\text{Length}}}}{2}} \right)$$

The body weight was recorded using laboratory balance every 2 days.

### Histology Analysis

The mice were sacrificed after PTT treatments, and tumors as well as other major organs were collected. Briefly, the organs were first fixed overnight in 4% formalin, and then embedded in paraffin. After deparaffinization in xylene twice, the tissue sections were sequent dehydrated by 100% alcohol twice (5 min once), 95% alcohol (2 min), 70% alcohol (2 min), and distilled water. For hematoxylin and eosin (H&E) staining, the sliced tumor sections were counterstained in hematoxylin solution (2%) and in eosin solution (0.5%), respectively.

### Hematology Analysis

Twenty healthy BALB/c mice were i.v. injected with RGD-PEG-Au@SiNRs (10 mg kg^−1^). Five mice at each time point (1, 7, 14, and 30 days) were sacrificed to collect blood for blood biochemistry and complete blood panel analysis. Healthy mice untreated were chosen as the control.

### Cytokines Detection

Serum samples were collected from treated mice at different time points and diluted for analysis. Interferon gamma (IFN-γ), interleukin-6 (IL-6), interleukin-2 (IL-2), and interleukin-1 (IL-1) were analyzed with ELISA kits according to vendors’ instructions (Biocentury).

## Results and Discussion

### Preparation and Characterization of Au@SiNRs

**Fig. 1:**
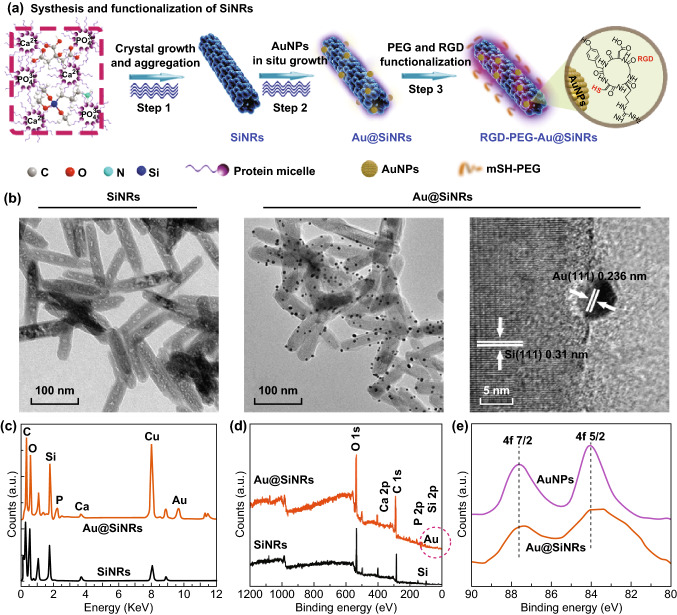
Schematic illustration of the synthetic procedure and characterization of Au@SiNRs. **a** The scheme of the fabrication of RGD-PEG-Au@SiNRs, including microwave synthesis of SiNRs, in situ growth of AuNPs on SiNRs, and surface modification of PEG and c(RGDyC). **b** TEM and HRTEM images of as-fabricated SiNRs and Au@SiNRs. **c** EDX spectra of Au@SiNRs (above) and SiNRs (below). **d** XPS spectra of the as-prepared Au@SiNRs (blue one) and SiNRs (black one). **e** Au 4f XPS spectra in AuNPs and Au@SiNRs. (Color figure online)

The fluorescent SiNRs with relatively strong and stable fluorescence (quantum yield of ~ 10.5%; fluorescence intensity preserves > 90% after 280-min UV irradiation or 100-day storage) were first prepared via our previously reported microwave method (Fig. S1) [30]. Afterward, AuNPs were grown in situ on their surface, producing the Au@SiNRs nanohybrid (Fig. 1a) [41, 48]. Figure 1b displays the TEM images of SiNRs and Au@SiNRs, both of which have the length of ~ 140 nm and the diameter of ~ 20 nm. Based on TEM images, the size of AuNPs was measured to be ~ 6.5 nm (Fig. S2). The high-resolution TEM (HRTEM) image demonstrates the high crystallinity of the as-prepared Au@SiNRs. Two well-defined lattice fringes with spaces of 0.236 and 0.31 nm were related to the (111) plane of Au and Si, respectively [6, 30]. Moreover, the presence of AuNPs was further confirmed by EDX and XPS analyses (Fig. 1c–e). Notably, high-resolution XPS spectrum of free AuNPs presents two peaks at 87.7 and 84.0 eV, corresponding to metallic Au 4f_7/2_ and 4f_5/2_, respectively, while there was a slight shift (~ 0.3 eV) in Au@SiNRs.

### Photophysical Properties of Au@SiNRs

**Fig. 2:**
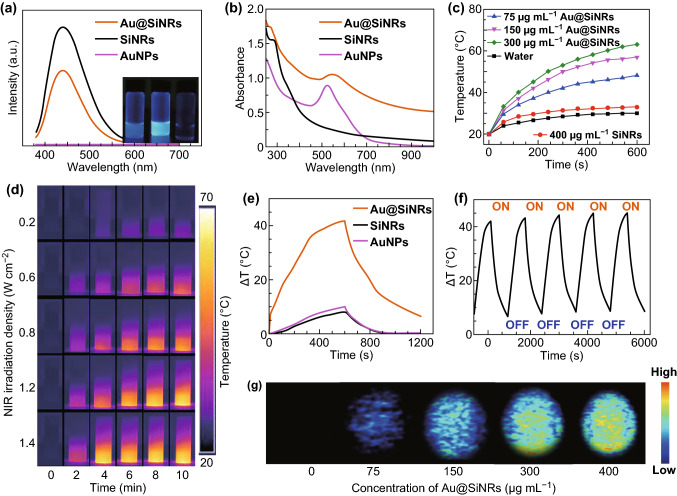
Photophysical properties of Au@SiNRs. **a** Photoluminescence spectra (λ_ex_ = 360 nm) and insert photographs of Au@SiNRs, SiNRs, and AuNPs from left to right. **b** UV–vis-NIR absorbance spectra of Au@SiNRs (300 μg mL^−1^), SiNRs (300 μg mL^−1^), and AuNPs (0.01 M). **c** Heating curves of Au@SiNRs solutions with different concentrations and SiNRs aqueous suspension (400 μg mL^−1^) at 1.2 W cm^−2^ for 600 s. **d** Infrared thermal images of Au@SiNRs suspensions (300 μg mL^−1^) irradiated with 808 nm laser at a variety of power densities for 0–10 min. **e** Heating–cooling curves of Au@SiNRs (300 μg mL^−1^), SiNRs (300 μg mL^−1^), and AuNPs (0.01 M). **f** The five-cycle photothermal profiles of Au@SiNRs (300 μg mL^−1^). **g** PA Imaging of Au@SiNRs with different concentrations

The photophysical properties of Au@SiNRs were investigated by PL and UV–vis-NIR spectroscopy. As shown in Fig. 2a, the Au@SiNRs show a distinguishable PL peak. As AuNPs is known able to quench fluorophores [26], the fluorescence intensity of Au@SiNPs was lower than that of SiNRs. Moreover, the fluorescence intensity of Au@SiNRs can be effectively regulated by the amount of AuNPs decorated on SiNRs (Fig. S3). Importantly, the as-fabricated Au@SiNRs show a high absorbance among the NIR region (700–1000 nm), accompanying with an extremely high mass extinction coefficient of 2.21 L g^−1^ cm^−1^ at 808 nm (Figs. 2b and S4). To investigate their potential as near-infrared hyperthermia agent, the photothermal properties of Au@SiNRs were systematically studied. As shown in Figs. 2c and S5, in contrast to water and free SiNRs, the Au@SiNRs solutions show a greatly rapid temperature rise in a concentration-dependent manner under 808-nm laser irradiation at a density of 1.2 W cm^−2^. A variety of power densities of 0.2, 0.6, 0.8, 1.2, and 1.4 W cm^−2^ were further examined to offer an optimized experimental condition (Figs. 2d and S6). It can be found that the temperature of Au@SiNRs solution (300 μg mL^−1^) has reached to a very high level (~ 57 °C) after 10-min irradiation at a 0.8 W cm^−2^ density.

Notably, the calculated photothermal conversion efficiency (η) of Au@SiNRs was as high as 43.9% in comparison with that of ~ 21% for gold nanorods (Figs. 2e and S7a) [49]. According to previous studies on metal-decorated SiNWs nanohybrids (AuNPs@SiNWs, PtNPs@SiNWs, and AgNPs@SiNWs) [48, 50, 51], it can be speculated that AuNPs on SiNRs would substantially enhance light conversion to heat, resulting in more pronounced photothermal performance of Au@SiNRs than free SiNRs or AuNPs. Moreover, Au@SiNRs also demonstrate a great photothermal stability, which is proved by the negligible change in their temperature elevation curve, absorption spectra, and morphology after five-cycle NIR laser irradiation (Figs. 2f and S8).

PA imaging has emerged as a novel and promising biomedical imaging modality, due to its significant improvement in imaging depth and spatial resolution in vivo [52, 53]. In PA imaging, ultrasound signals will be generated when tissues or contrast probes absorb and convert the delivered energy into heat. As described above, as-prepared Au@SiNRs have an extremely high photothermal conversion efficiency, providing possibilities to be used as contrast agents for PA imaging. As shown in Fig. 2g, Au@SiNRs solutions show a concentration-dependent PA signal intensity. The quantitative analysis further demonstrates there is a positive linear relationship between signal intensities and concentrations (Fig. S9). In contrast, no PA signal was detected for SiNRs or AuNPs under identical conditions (Fig. S10). These findings demonstrate the potential of Au@SiNRs as a multimodal contrast agent with tunable fluorescence signal, high photothermal conversion efficiency and good photostability.

### In vitro Assessment of Biocompatibility, Targeted imaging, and Photothermal Effect

**Fig. 3:**
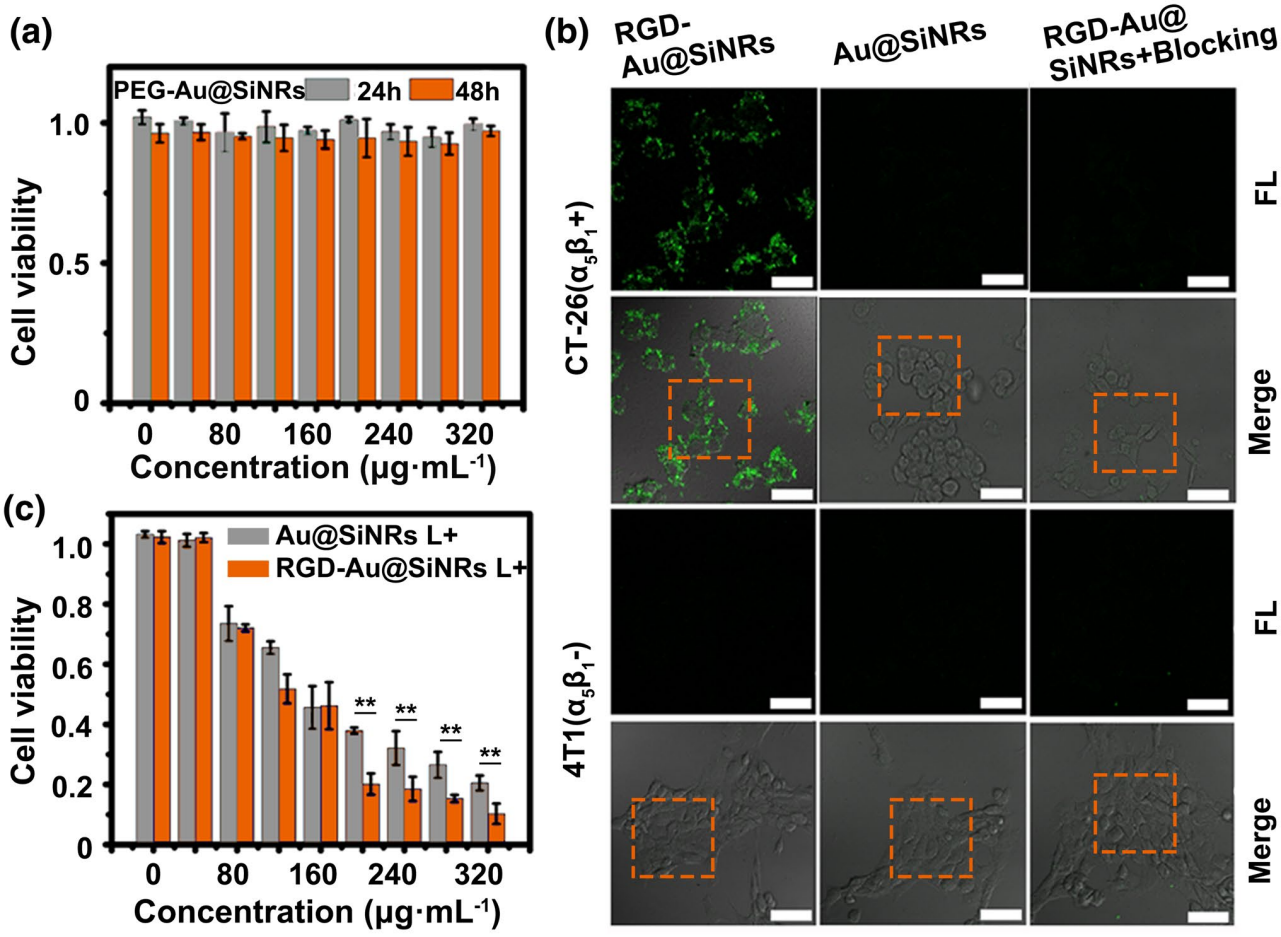
Assessment of the biocompatibility, targeted imaging, and photothermal effect in vitro. **a** Cytotoxicity of PEG-Au@SiNRs. **b** LSCM images of CT-26 and 4T1 cells after incubation with RGD-Au@SiNRs (blocking with free peptides or not) or Au@SiNRs for 2 h at 37 °C. Scale bars, 25 μm. **c** Cell viability of CT-26 cells, which were first incubated with RGD-Au@SiNRs or Au@SiNRs for 4 h and then irradiated by an 808-nm laser (0.8 W cm^−2^) for 5 min as mean ± SD (*n* = 3). Asterisk (**) indicates *p *< 0.01

To improve their biocompatibility, the as-fabricated Au@SiNRs were conjugated with thiol-terminated methyl-polyethylene glycol (mPEG-SH) via Au–S bonds, producing the PEGylated Au@SiNRs (PEG-Au@SiNRs). The zeta potential of PEG-Au@SiNRs was determined as − 10.3 ± 1.2 mV, in contrast to that of 1.3 ± 0.5 mV for SiNRs (Fig. S11). The evaluation of the effects of PEG-Au@SiNRs on cell viability demonstrates their low-/non-toxic toward CT-26 cells (murine colorectal carcinoma cell line) during 24- or 48-h incubation with gradient concentrations (0 ~ 320 μg mL^−1^) (Fig. 3a). On the other hand, to enhance their performance on tumor diagnosis and therapy, the cyclic peptides ligands c(RGDyC) were chosen as the active targeting moiety because of their strong specific binding capacity to integrin receptors (α_v_β_3_ and α_5_β_1_) overexpressing on cancer and angiogenic endothelial cells [42, 54]. Through Au–S bonds, thiol-terminated c(RGDyC) peptides can be easily linked to the surface of PEG-Au@SiNRs, yielding RGD-PEG-Au@SiNRs. Significantly, the modification of PEG and RGD molecules has no effect on the photophysical properties of Au@SiNRs, that is, like Au@SiNRs, PEG-Au@SiNRs, and RGD-PEG-Au@SiNRs have similar UV and PL spectra, PA signals, and photothermal effects (Fig. S12). The photothermal conversion efficiency of RGD-PEG-Au@SiNRs was calculated to be 36.1%, slightly lower than that of Au@SiNRs (Fig. S7b). Moreover, the RGD-PEG-Au@SiNRs show a good stability in water, PBS, and RPMI-1640 culture medium during 7-day storage (Fig. S13). To simplify the writing, the PEG-Au@SiNRs and RGD-PEG-Au@SiNRs were abbreviated as Au@SiNRs and RGD-Au@SiNRs in the following sections.

The biological activity of the prepared RGD-Au@SiNRs was verified in vitro. As shown in Fig. 3b, the RGD-Au@SiNRs-treated CT-26 cells (integrin α_5_β_1_ positive) exhibit strong fluorescence, while only feeble fluorescence could be observed from those treated with Au@SiNRs. Additionally, the fluorescence intensity of CT-26 cells treated with RGD-Au@SiNRs was significantly reduced when the integrin receptors were blocked with free RGD peptides. In contrast, integrin α_5_β_1_ negative cells (4T1 cells, the murine breast carcinoma cancer cell line) express weak fluorescence signal no matter how they were treated with Au@SiNRs, RGD-Au@SiNRs or blocked with RGD peptides before the treatment of RGD-Au@SiNRs. The results were supported by the different average fluorescent intensities quantified by the LSCM software (Fig. S14). Furthermore, the time-dependent cellular uptake of RGD-Au@SiNRs by CT-26 cells was investigated by flow cytometry, which showed that after incubation for only 0.5 h, more than 80% cells have uptaken the nanoagents (Fig. S15). These data confirm the targeting ability of RGD-Au@SiNRs to some integrin (α_v_β_3_ and α_5_β_1_), which is in accordance with the previous studies [55, 56].

Encouraged by the good photothermal efficacy and biocompatibility of Au@SiNRs, the PTT effect was first evaluated in vitro. After incubation with different agents (PBS, AuNPs, SiNRs, Au@SiNRs, and RGD-Au@SiNRs) for 4 h, the CT-26 cells were washed and non- or irradiated with a NIR laser (808 nm, 0.8 W cm^−2^) for 5 min. The efficacy of photothermal therapy of Au@SiNRs and RGD-Au@SiNRs was quantitatively assessed by measuring the cell viability via MTT method. Both Au@SiNRs and RGD-Au@SiNRs show a dose-dependent PTT efficacy, while RGD-Au@SiNRs had a better ablation effect than that of Au@SiNRs (Fig. 3c). The live/dead [calcein-AM/propidium iodide (PI)] staining was also applied to visually evaluate the cell viability, where the green and red fluorescence indicates the live and dead cells, respectively. The results clearly demonstrate the treatment of Au@SiNRs or RGD-Au@SiNRs would induce cells death when the cells were irradiated with a laser, while AuNPs and SiNRs have no affect on the viability of CT-26 cells at the tested concentrations (Fig. S16). In addition, the efficacy of RGD-Au@SiNRs is better due to the targeting ability of c(RGDyC) peptides. In contrast, without NIR laser irradiation, AuNPs, SiNRs, Au@SiNRs, and RGD-Au@SiNRs have negligible effect on the viability of CT-26 cells at the tested concentrations.

### In Vivo Tumor-targeted Multimodal Imaging

**Fig. 4:**
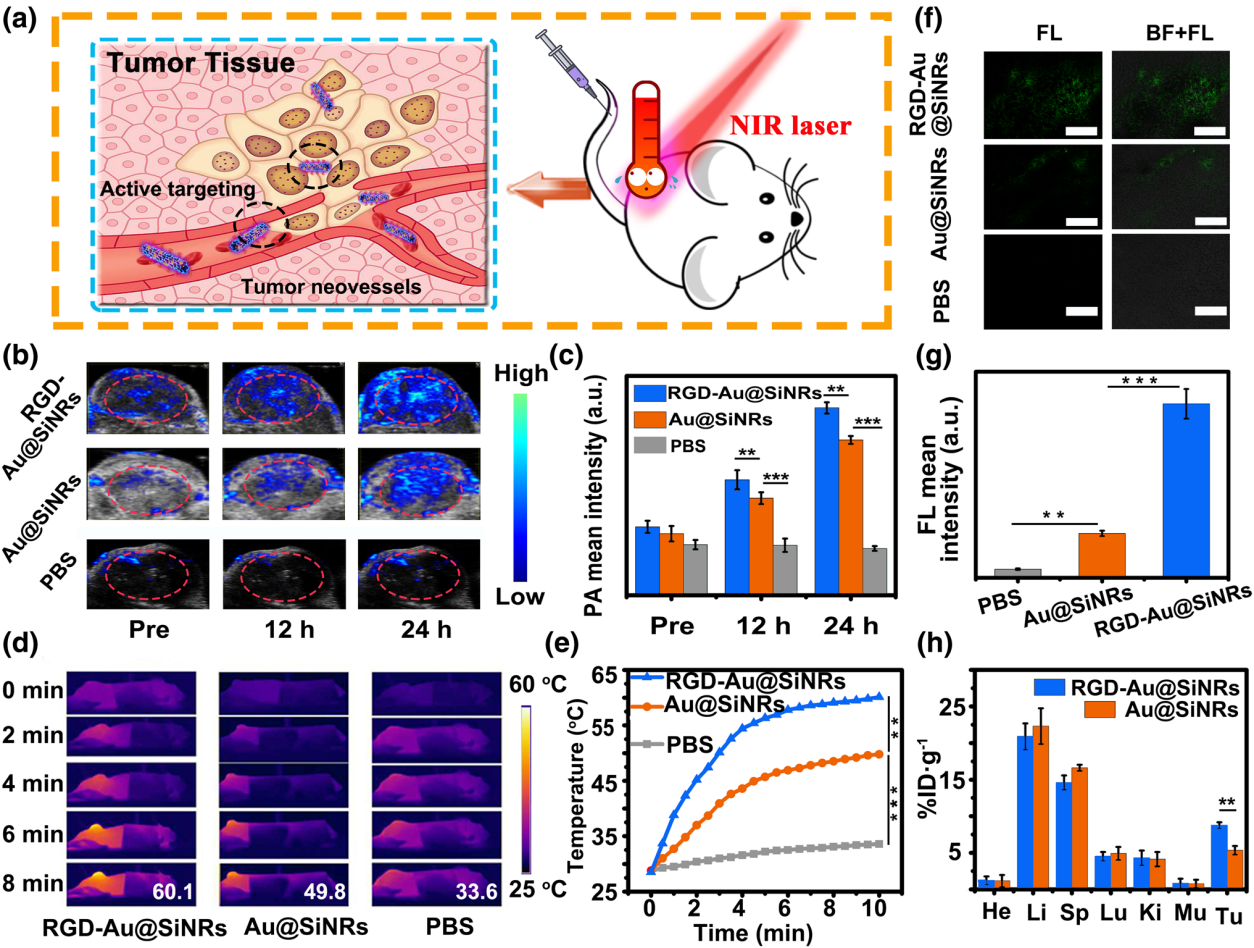
Tumor-targeted multimodal imaging in vivo. **a** Schematic illustration of the active targeting of RGD-Au@SiNRs. **b** PA imaging and **c** the corresponding PA signal intensity of tumor regions of CT-26 tumor-bearing mice untreated and treated with RGD-Au@SiNRs, Au@SiNRs, or PBS for 12 and 24 h. **d** Infrared thermal mapping images, and **e** corresponding temperature change of tumor regions of CT-26 tumor-bearing mice irradiated with an 808-nm laser (0.8 W cm^−2^) for different times (0 ~ 8 min, time interval: 30 s) at 24-h post-administration with RGD-Au@SiNRs, Au@SiNRs, or PBS. **f** LSCM images of tumor sections at 24-h post-injection of PBS, Au@SiNRs, or RGD-Au@SiNRs. Scale bars, 100 μm, and **g** corresponding quantitative analysis of the fluorescence intensity. **h** The bio-distribution of RGD-Au@SiNRs and Au@SiNRs measured by ICP-OES at 24-h post-administration. Asterisk (**) indicates *p *< 0.01; (***) means *p* < 0.001

Next, the as-prepared RGD-Au@SiNRs were employed as the contrast agent for tumor-targeted PA/PL/PTT triple-modal imaging in vivo. The CT-26 tumor-bearing mice were first i.v. injected with RGD-Au@SiNRs, Au@SiNRs, or PBS; then, the tumor regions were detected through different imaging systems (Fig. 4a). As shown in Fig. 4b, the cross-sectional PA signals of tumor regions reached to a high level after the injection with RGD-Au@SiNRs or Au@SiNRs for 24 h. For Au@SiNRs, the results should be attributed to the distinct enhanced permeability and retention (EPR) effect of tumors [40] and the nanoEL (nanoparticle-induced endothelial leakiness) effect [57–59]. Comparatively, in terms of RGD-Au@SiNRs, the active targeting plays a positive effect on their accumulation to tumors, as the intensity of PA signal generated by RGD-Au@SiNRs is obviously higher than that generated by Au@SiNRs (Fig. 4c). The infrared thermal mapping was then utilized to directly image the temperature changes at the tumor site of different treatment groups after NIR irradiation. According to above-mentioned results of PA and fluorescence imaging, tumor-bearing mice were irradiated with an 808-nm NIR laser (0.8 W cm^−2^) at 24-h post-injection of RGD-Au@SiNRs, Au@SiNRs, and PBS for different times (0 ~ 8 min). As shown in Fig. 4d, e, after 8-min irradiation, the temperature of tumor region reached up to 60.1 °C in RGD-Au@SiNRs-treated group, while the Au@SiNRs and PBS-treated ones increased to 49.8 and 33.6 °C, respectively. According to the positive relationship between the temperature rise and concentration, the results suggest that the tumors in RGD-Au@SiNRs-treated group have taken up more nanostructures than that in Au@SiNRs-treated group. Additionally, fluorescence images of tumor sections show that the tumor site of RGD-Au@SiNRs-treated group had higher fluorescence signal than that of Au@SiNRs (Fig. 4f, g), further indicating better tumor-targeting capability of RGD-Au@SiNRs.

Through PA imaging of the bladders of mice i.v. injected with RGD-Au@SiNRs, it was found that the signal of bladders reached to the highest level at 8-h post-injection, and then gradually decreases (Fig. S17a, b), while the blood circulation half-life of i.v. injected RGD-Au@SiRNs was measured to be ~ 4.0 h (Fig. S17c). In order to quantify the bio-distribution of our nanostructures in vivo, the gold element-based ICP-OES analysis was employed at 24-h post-injection. Significantly, the tumor uptake of RGD-Au@SiNRs was measured to be 8.74% ID g^−1^, which was obviously higher than that of Au@SiNRs (5.32% ID g^−1^) (*p* < 0.01) (Fig. 4h). Meanwhile, high levels of Au content were observed in the liver and spleen, which were reticuloendothelial systems (RES) responsible for the metabolism and clearance of nanorods [60, 61]. Thus, RGD-Au@SiNRs have a remarkable PA/PL/PTT triple-modal imaging capability and an obvious tumor-homing effect.

### Antitumor Effect and Biosafety Assessment

**Fig. 5:**
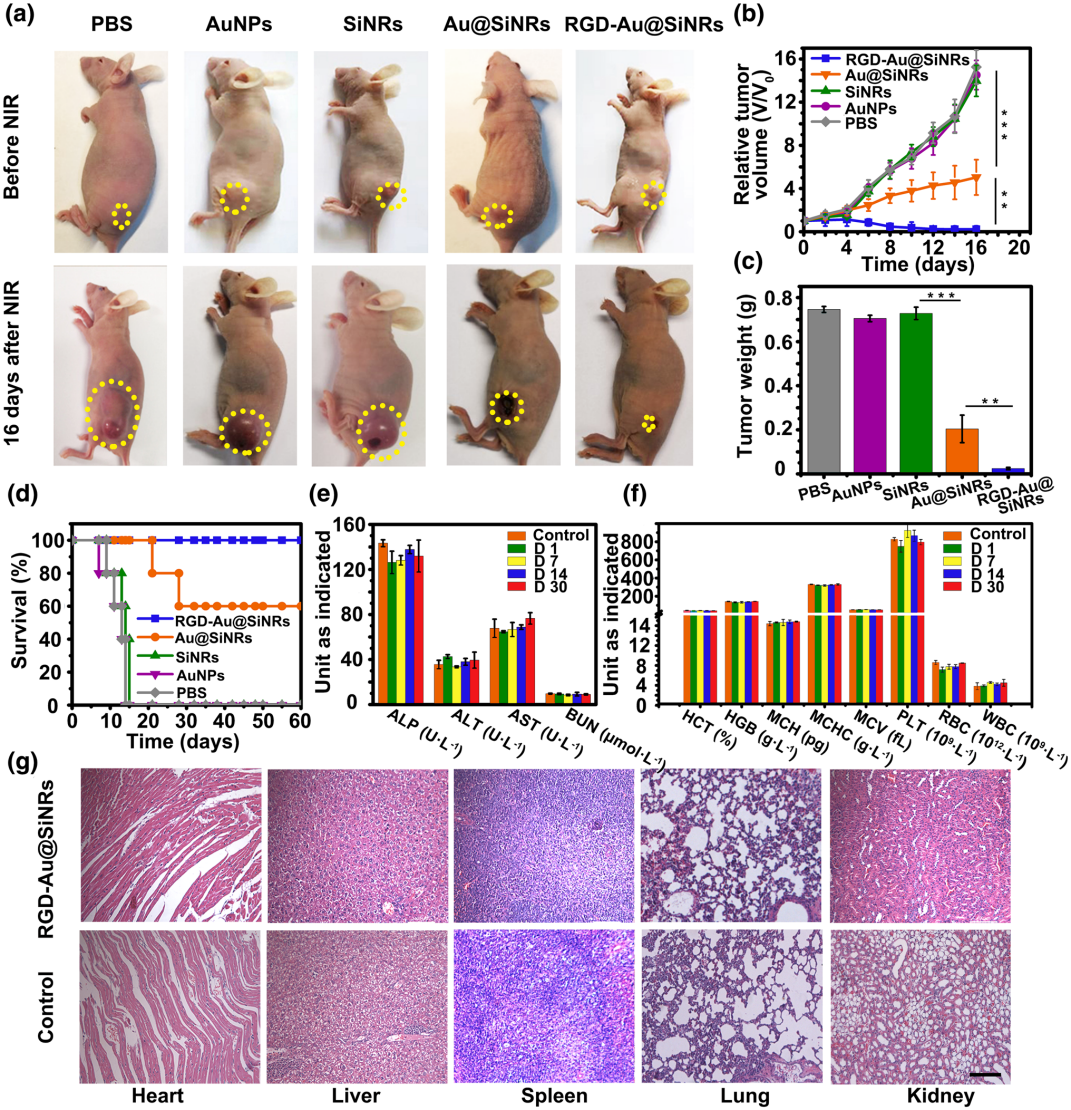
Photothermal therapy and safety assessment. **a** Photos of representative mice before and after the treatment with different agents and NIR irradiation. **b** Growth curves of tumor volumes of mice groups with NIR irradiation. **c** Weight of the excised tumors from the PTT-treated mice. **d** Survival curves of PTT-treated mice. **e** Serum biochemistry data including alkaline phosphatase, alanine aminotransferase, and aspartate aminotransferase, and blood urea nitrogen levels of control and RGD-Au@SiNRs-treated healthy mice. **f** Complete blood counts: hematocrit, hemoglobin, mean corpuscular hemoglobin, mean corpuscular hemoglobin concentration, mean corpuscular volume, blood platelets, red blood cells, blood levels of white blood cells, and platelets of control and RGD-Au@SiNRs-treated healthy mice. **g** H&E staining of various organ tissues harvested from tumor-bearing mice at the end of treatment. Asterisk (**) indicates *p *< 0.01; (***) means *p* < 0.001

With the guidance of multimodal imaging and obvious tumor-homing effect, the PTT efficacy of Au@SiNRs was further investigated in vivo. After CT-26, tumor-bearing mice were i.v. injected with PBS, AuNPs, SiNRs, Au@SiNRs, or RGD-Au@SiNRs (SiNRs: 20 mg kg^−1^; AuNPs: 0.62 mg kg^−1^) for 24 h, and the tumor regions were non- or irradiated under 808-nm NIR laser at 0.8 W cm^−2^ for 8 min with an interval 30 s. Without NIR laser irradiation, the mice treated with PBS, AuNPs, SiNRs, Au@SiNRs, or RGD-Au@SiNRs show a similar and rapid tumor growth during 16 days, indicating that AuNPs, SiNRs, or Au@SiNRs alone have little influence on tumor growth (Figs. S18 and S19). Once the mice were irradiated with NIR laser, the treatment with Au@SiNRs or RGD-Au@SiNRs significantly reduced the tumor growth, while AuNPs and SiNRs still had negligible effect (Fig. 5a–d). Typically, at 16-day posttreatment, the tumors totally disappeared in mice treated with RGD-Au@SiNRs and NIR laser, where only a small scar was left (Fig. 5a). The tumor volume and weight measurement shows that with NIR laser, administration of Au@SiNRs or RGD-Au@SiNRs could obviously inhibit tumor growth, while RGD-Au@SiNRs had a better efficacy and regressed the tumor growth from the fourth day of posttreatment (Fig. 5b, c). Reasonably, it can be found that the overall survival time of mice treated with Au@SiNRs or RGD-Au@SiNRs was prolonged in comparison with that of three control groups (PBS-, AuNPs-, and SiNRs-treated mice) (Fig. 5d). Significantly, all the mice treated with RGD-Au@SiNRs remained alive, and no distinct tumor recurrence was observed during our 60-day investigation.

This novel SiNRs-based imaging-guided NIR hyperthermia agent shows non-/low toxic effects on mice. During the therapeutic period, the mouse body weight of all groups showed negligible drop with or without irradiation (Fig. S20). Moreover, as shown in Fig. 5e, f, several classes of dominant serum biochemical markers, and blood count parameters were all normal at different time points (1, 7, 14, and 30 days) at post-intravenous injection of RGD-Au@SiNRs. Histology analysis of dominant organs also showed no obvious pathological abnormalities or lesions (Figs. 5g and S21). Noteworthy, there were temporary rises in IL-6 and IFN-γ after 4- or 24-h treatment with RGD-Au@SiNRs, while the levels of both of them decrease to normal within 48 h (Fig. S22).

## Conclusions

In summary, we present a kind of silicon-based multifunctional nanostructures, i.e., the Au@SiNRs, which are exploited as high-quality theranostic agent for multimodal imaging-guided cancer therapy. The as-prepared Au@SiNRs featuring high photothermal conversion efficacy and good photothermal stability could serve as multifunctional agents, enabling PA- and infrared thermal imaging-guided PTT. A facile surface modification makes the fabricated RGD-PEG-Au@SiNRs having an obvious tumor-homing effect, resulting in an efficient therapeutic effect on tumors after a systemic administration. Moreover, no appreciable toxicity was observed after intravenous injection of Au@SiNRs into mice. Given that silicon nanostructures have several intrinsic advantages like abundant source and biodegradability, the developed Au@SiNRs may act as practical nanotheranostic agents for imaging-guided cancer treatment, holding high prospects in the era of personalized medicine.

## Electronic Supplementary Material

Below is the link to the electronic supplementary material.

Supplementary material 1 (PDF 2239 kb)
