# Correction to: Fluorescent Silicon Nanorods‑Based Nanotheranostic Agents for Multimodal Imaging‑Guided Photothermal Therapy

**DOI:** 10.1007/s40820-019-0318-5

**Published:** 2019-10-15

**Authors:** Mingyue Cui, Sangmo Liu, Bin Song, Daoxia Guo, Jinhua Wang, Guyue Hu, Yuanyuan Su, Yao He

**Affiliations:** Laboratory of Nanoscale Biochemical Analysis Institute of Functional Nano & Soft Materials (FUNSOM), and Jiangsu Key Laboratory for Carbon-Based Functional Materials & Devices, Soochow University, 199 Ren’ai Road, Suzhou, 215123 Jiangsu People’s Republic of China

## Correction to: Nano-Micro Lett. (2019) 11:73 10.1007/s40820-019-0306-9

In the original publication figures 3b, 5a, 5g are incorrectly published and the scale bars in figures 4b and 4d have not appeared.

**Fig. 3:**
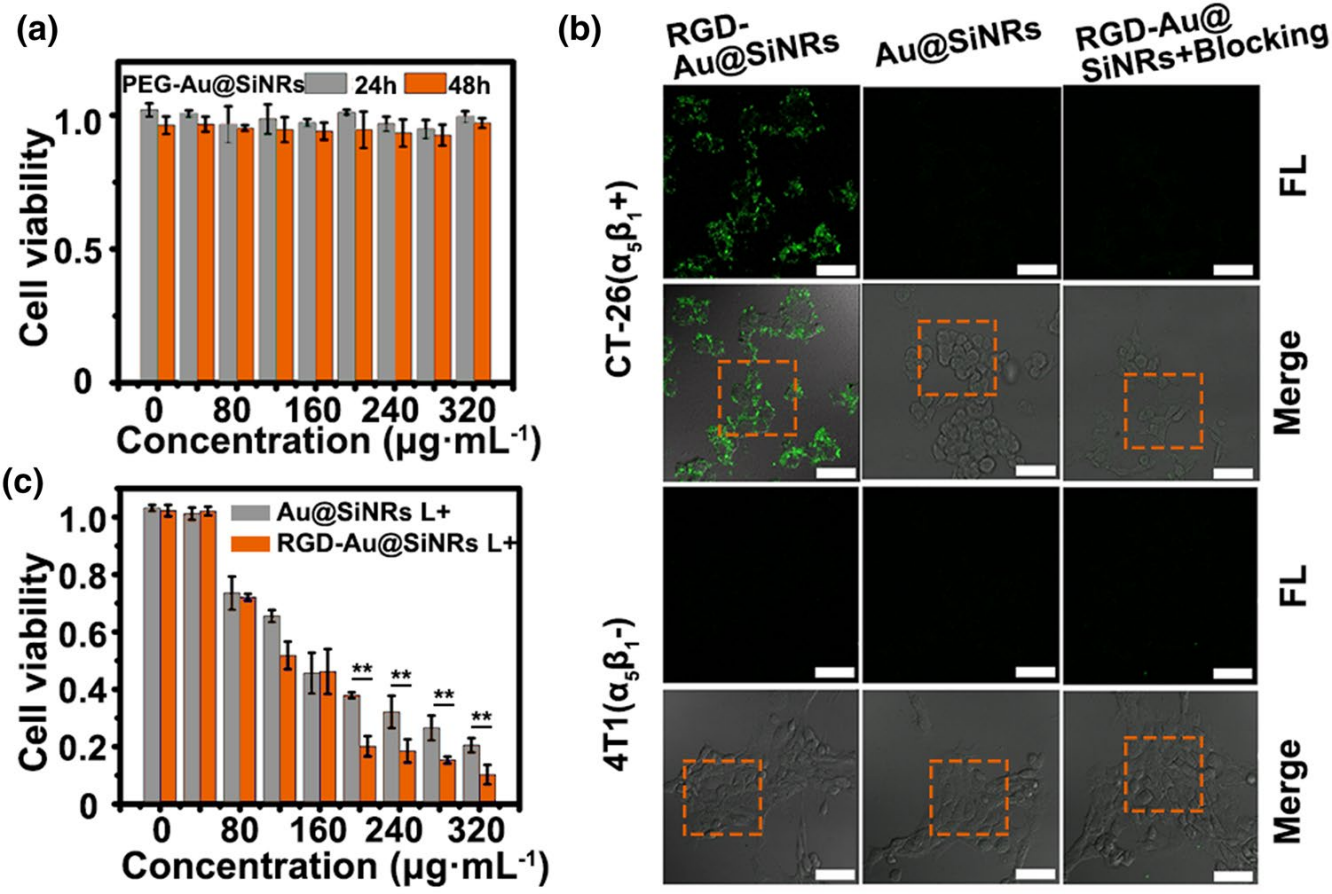
Assessment of the biocompatibility, targeted imaging, and photothermal effect in vitro. **a** Cytotoxicity of PEG-Au@SiNRs. **b** LSCM images of CT-26 and 4T1 cells after incubation with RGD-Au@SiNRs (blocking with free peptides or not) or Au@SiNRs for 2 h at 37 °C. Scale bars, 25 μm. **c** Cell viability of CT-26 cells, which were first incubated with RGD-Au@SiNRs or Au@SiNRs for 4 h and then irradiated by an 808-nm laser (0.8 W cm^−2^) for 5 min as mean ± SD (*n *= 3). Asterisk (**) indicates *p *< 0.01

**Fig. 4:**
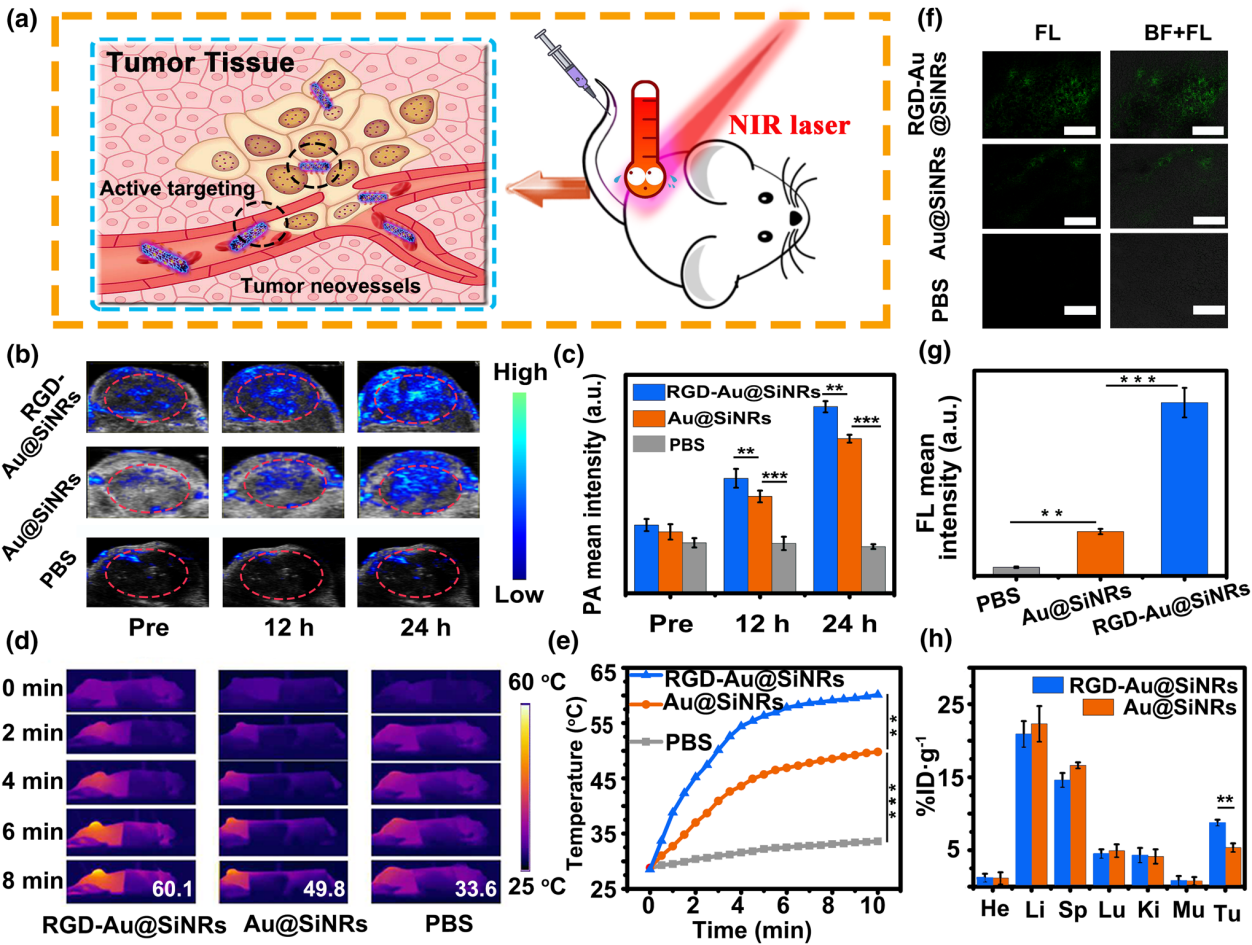
Tumor-targeted multimodal imaging in vivo. **a** Schematic illustration of the active targeting of RGD-Au@SiNRs. **b** PA imaging and **c** the corresponding PA signal intensity of tumor regions of CT-26 tumor-bearing mice untreated and treated with RGD-Au@SiNRs, Au@SiNRs, or PBS for 12 and 24 h. **d** Infrared thermal mapping images, and **e** corresponding temperature change of tumor regions of CT-26 tumor-bearing mice irradiated with an 808-nm laser (0.8 W cm^−2^) for different times (0–8 min, time interval: 30 s) at 24 h post-administration with RGD-Au@SiNRs, Au@SiNRs, or PBS. **f** LSCM images of tumor sections at 24 h post-injection of PBS, Au@SiNRs, or RGD-Au@SiNRs. Scale bars, 100 μm, and **g** corresponding quantitative analysis of the fluorescence intensity. **h** The bio-distribution of RGD-Au@SiNRs and Au@SiNRs measured by ICP-OES at 24 h post-administration. Asterisk (**) indicates *p *< 0.01; (***) means *p *< 0.001

**Fig. 5:**
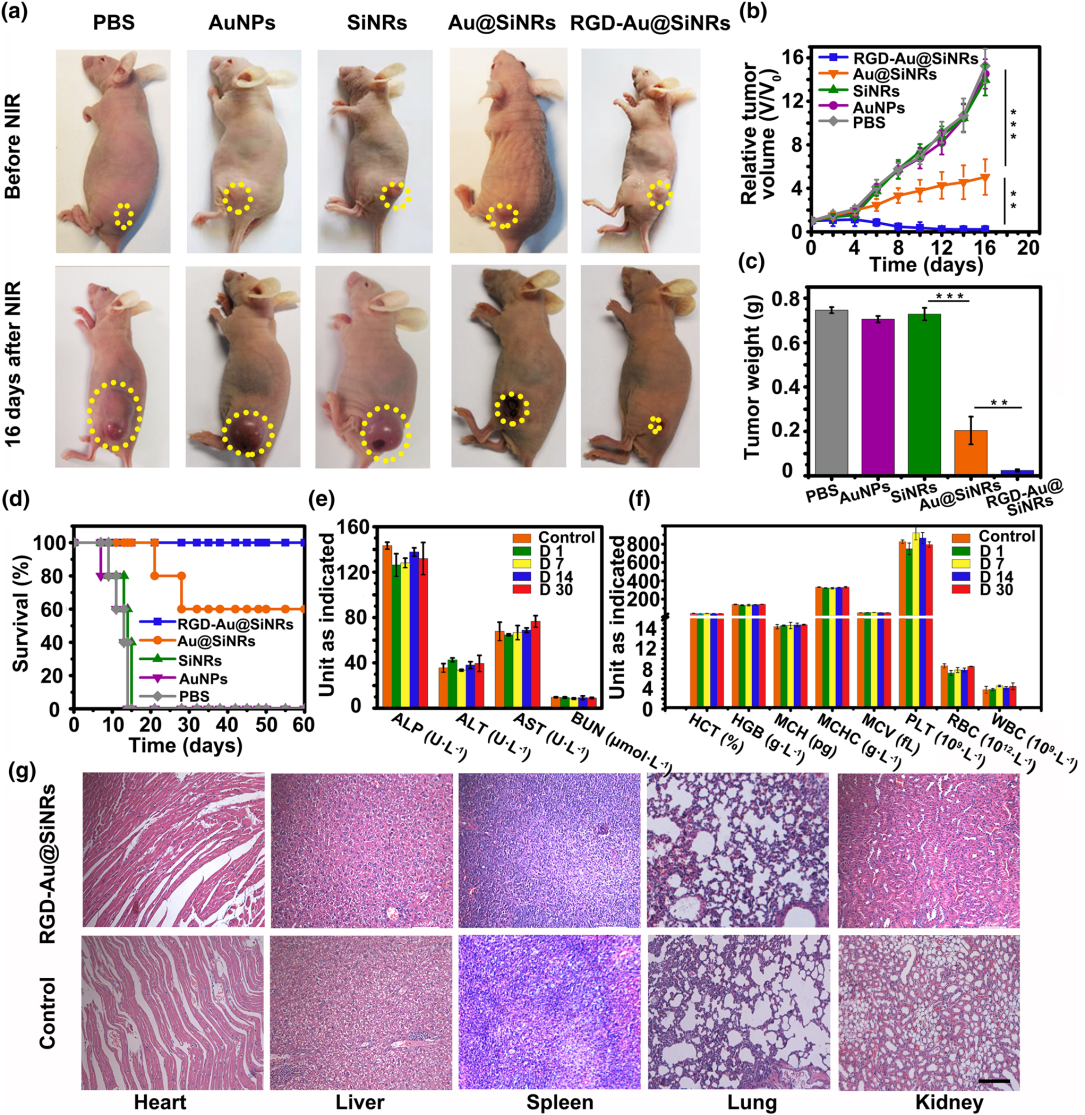
Photothermal therapy and safety assessment. **a** Photographs of representative mice before and after the treatment with different agents and NIR irradiation. **b** Growth curves of tumor volumes of mice groups with NIR irradiation. **c** Weight of the excised tumors from the PTT-treated mice. **d** Survival curves of PTT-treated mice. **e** Serum biochemistry data including alkaline phosphatase, alanine aminotransferase, and aspartate aminotransferase, and blood urea nitrogen levels of control and RGD-Au@SiNRs-treated healthy mice. **f** Complete blood counts: hematocrit, hemoglobin, mean corpuscular hemoglobin, mean corpuscular hemoglobin concentration, mean corpuscular volume, blood platelets, red blood cells, blood levels of white blood cells, and platelets of control and RGD-Au@SiNRs-treated healthy mice. **g** H&E staining of various organ tissues harvested from tumor-bearing mice at the end of treatment. Asterisk (**) indicates *p *< 0.01; (***) means *p *< 0.001

The correct figures are provided in this correction.

The original article has been corrected.

