# Supplementary material for: Fluorescent Silicon Nanorods-Based Nanotheranostic Agents for Multimodal Imaging-Guided Photothermal Therapy
Source: Nanomicro Lett. 2019 Sep 9;11:73. doi: 10.1007/s40820-019-0306-9 (PMC7770883; doi:10.1007/s40820-019-0306-9)
Supplement: Supplementary file 1 — Supplementary material 1 (PDF 2239 kb) [file 40820_2019_306_MOESM1_ESM.pdf]

Nano-Micro Letters

SUPPLEMENTAL INFORMATION FOR

Fluorescent Silicon Nanorods-Based Nanotheranostic Agents for Tumor-Targeted Multimodal Imaging-Guided Photothermal Therapy

Mingyue Cui[1], Sangmo Liu[1], Bin Song[1], Daoxia Guo[1], Jinhua Wang[1], Guyue Hu[1], Yuanyuan Su[1],* and Yao He[1]*

Laboratory of Nanoscale Biochemical Analysis, Institute of Functional Nano & Soft Materials (FUNSOM), and Jiangsu Key Laboratory for Carbon-Based Functional Materials & Devices, Soochow University, 199 Ren'ai Road, Suzhou, 215123, Jiangsu, PR China

✉ Yuanyuan Su, suyuanyuan@suda.edu.cn; Yao He, yaohe@suda.edu.cn

Additional data

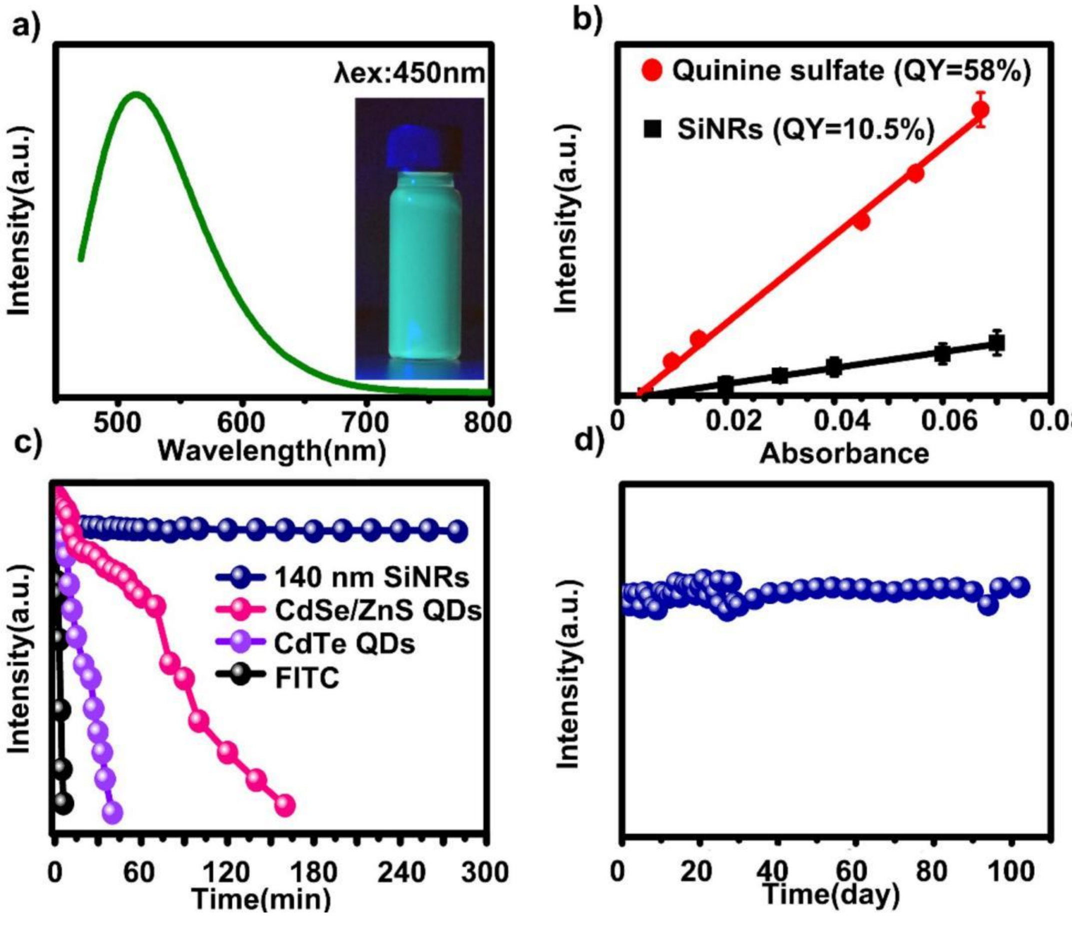

Fig. S1 Fluorescent characteristics and photo-stability of SiNRs. a PL spectra and photograph of SiNRs (Excitation wavelength (λ_{ex}) = 450 nm). **b** PLQYs measurements of the SiNRs and **c** Photostability comparison of aqueous solutions ofFITC, CdTe QDs, CdSe/ZnS QDs under continuous UV irradiation (450 W xenon lamp) for serial times. **d** The as-prepared aqueous samples of the SiNRs retain nearly identical fluorescent intensity during 100 days of storage.

As shown in Fig. S1, the the aqueous solution of the 140 nm SiNRs shows strong green fluorescence (photoluminescence quantum yield (PLQY): 10.5 %) under 450 nm irradiation. Notably, the as-prepared SiNRs preserve extremely stable optical properties even after high-power UV irradiation and long-term storage. The SiNRs sample maintains bright fluorescence during 280 min of UV irradiation. In sharp contrast, for the samples ofFITC, CdTe QDs, and CdSe/ZnS QDs, their fluorescence is strong in the beginning but gradually vanishes as irradiation time increases. In addition to robust photostability, the PL intensity ofSiNRs is stable during the 100-day-storage in ambient environment without any special protection.

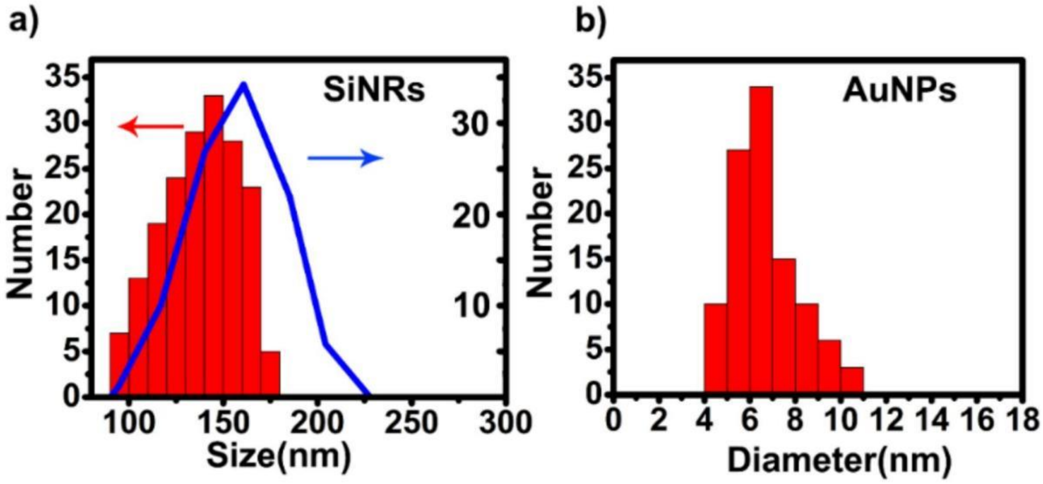

Fig. S2 Size distribution of Au@SiNRs and the decorated AuNPs. a The size distribution analysis of as-prepared SiNRs determined by TEM (red histogram) and DLS spectra (blue curves). **b** The size distribution histogram ofAuNPs determined by TEM images ofAu@SiNRs.

The size measurement based on TEM images ofas-prepared SiNRs confirms a narrow size distribution, and the length of the SiNRs is ~140 nm, which is close to the result of ~162 nm measured by DLS. The size distribution histogram ofAuNPs, determined by measuring more than 100 particles from TEM images of Au@SiNRs, indicates that the average size and standard deviation of the AuNPs is 6.5 ± 2.4 nm.

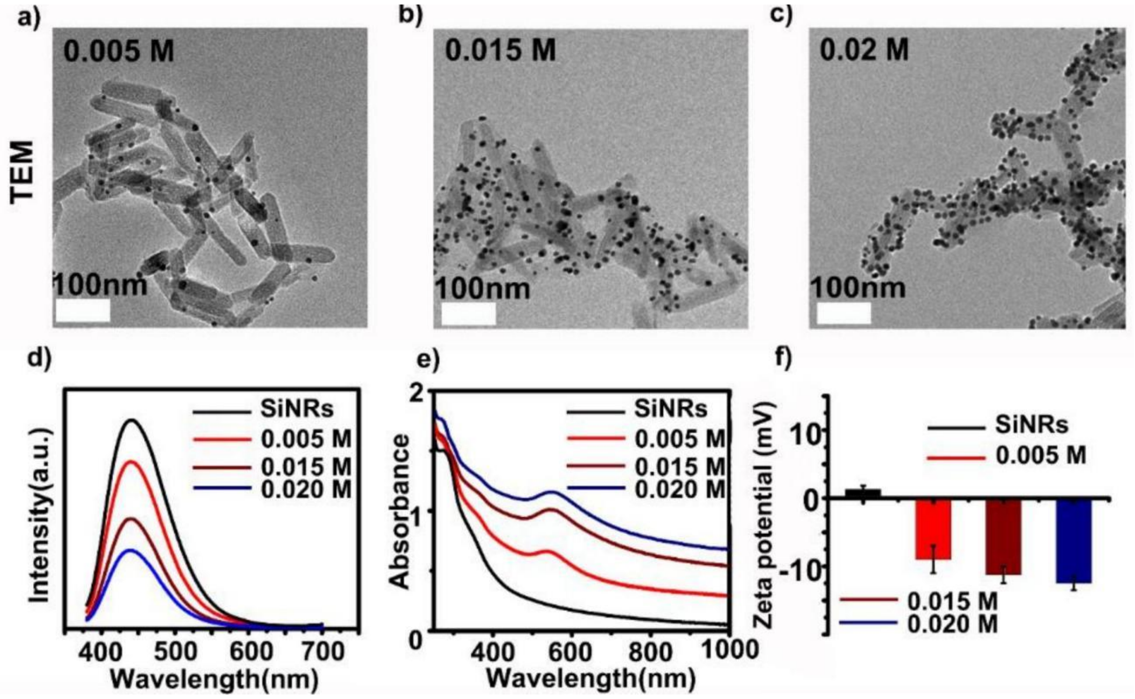

Fig. S3 Effect of surface decoration of AuNPs on fluorescence, UV absorption, Zeta potential and morphology of SiNRs. TEM images of the Au@SiNRs synthesized with $HAuCl_4$ concentrations of **a** 0.005 M, **b** 0.015 M, **c** 0.02 M. **d** The fluorescence spectra under 360 nm excitation and **e** corresponding UV absorption spectra. **f** Zeta potential of SiNRs and Au@SiNRs with different $HAuCl_4$ concentrations.

As presented in Fig. S3 (a-c), with the increase of the concentration of $HAuCl_4$ (0.005 ~0.020 M), more AuNPs were grown on the surface of each SiNRs. Meanwhile, more AuNPs decorated on the surface, fluorescence was more severely quenched and NIR absorbance was higher. Additionally, decoration ofAuNPs induces the surface charge of as-prepared Au@SiNRs to be negative.

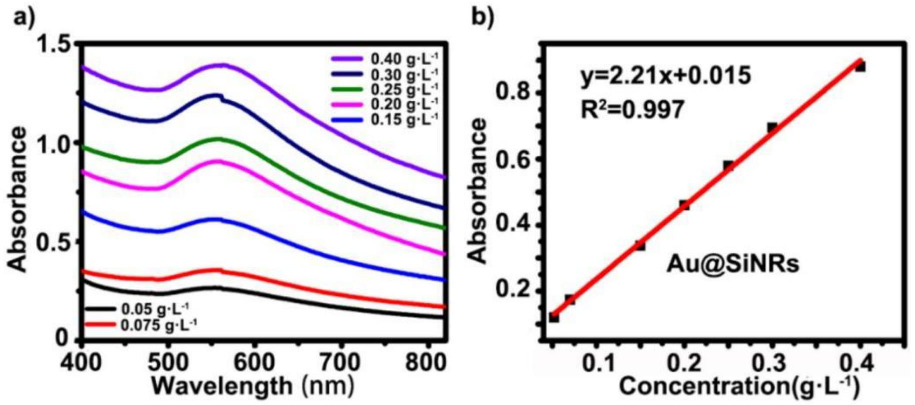

Fig. S4 Absorbance at 808 nm vs. concentrations of Au@SiNRs. a UV absorption spectra of Au@SiNRs with different concentration (0.05 ~ 0.4 g L^{-1}). **b** Absorbance at 808 nm vs concentration of Au@SiNRs. Solid line is the linear fit using the analysis tool in Origin software with R^2 = 0.997.

There is a good linear correlation between the absorbance at 808 nm and the concentration of Au@SiNRs. The mass extinction coefficient at 808 nm can be calculated according to Lambert-Beer law (A/L = αC, where A is the absorption intensity, L is the length of the cuvette, C is the concentration, and α is the extinction coefficient). In our following studies, the concentrations of Au@SiNRs and SiNRs were determined through their absorbance at 808 nm.

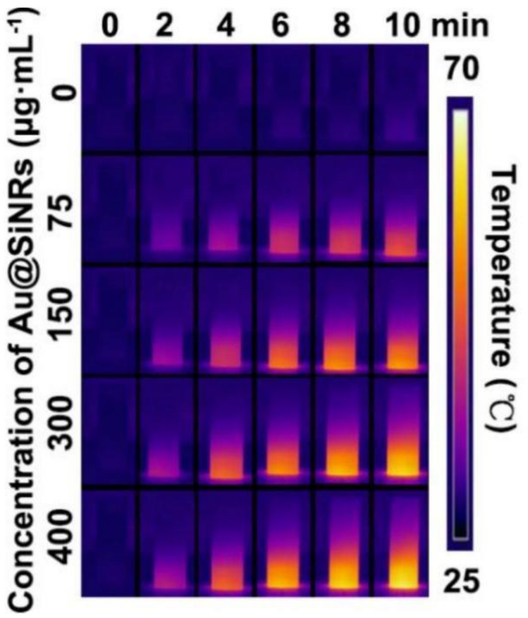

Fig. S5 Infrared thermal images for concentration-dependent manner under the 808-nm laser irradiation (1.2 W·cm^{-1}) of Au@SiNRs. Pure water was used as negative control.

An obvious concentration-dependent increase was observed in temperature that is a higher concentration would lead to a quicker increase of the temperature.

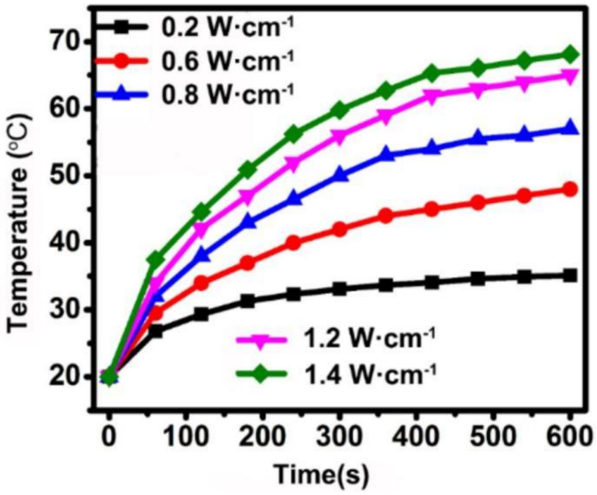

Fig. S6 Heating curves of Au@SiNRs solutions (300 μg·mL^{-1}) under 808 nm laser irradiation with different power densities (i.e., 0.2, 0.6, 0.8, 1.2, 1.4 W·cm^{-2}) for 600s.

Significantly, a power density-dependent increase was also observed in temperature. Typically, after 10-min laser irradiation with power densities of 0.2, 0.6, 0.8, 1.2, and 1.4 W·cm^{-1}, the sample temperatures increased to ~35, 48, 57, 65, and 68°C, respectively.

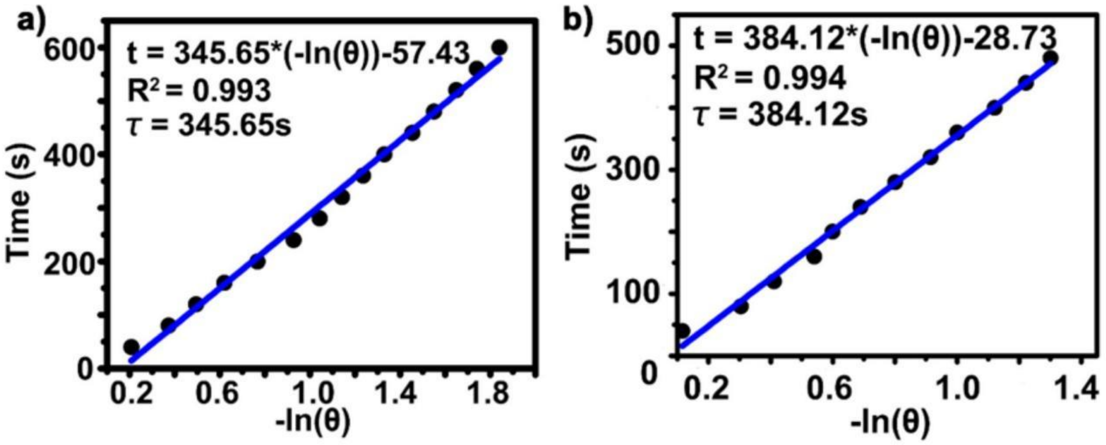

Fig. S7 Plot and linear fit of time versus negative natural logarithm of the temperature increment for the cooling rate of **a** Au@SiNRs and **b** RGD-PEG-Au@SiNRs suspensions.

Time constant for heat transfer from the system is determined to be τ_s = 345.65 and 384.12 s for Au@SiNRs and RGD-PEG-Au@SiNRs respectively by applying the linear time data from the cooling period (after 600 s) versus negative natural logarithm of driving force temperature, which is obtained from the cooling stage.

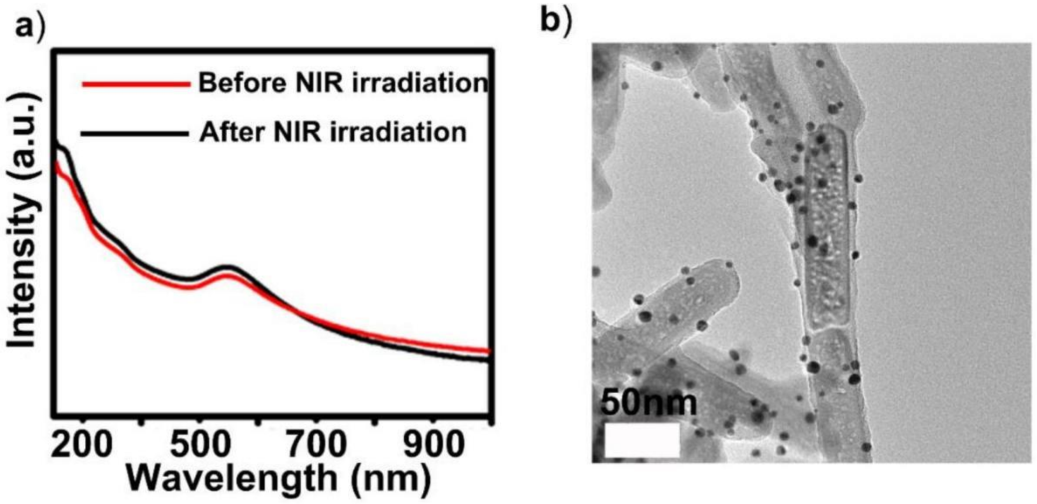

Fig. S8 Absorbance and morphology change of Au@SiNRs after NIR irradiation. **a** The UV-Vis spectra of Au@SiNRs before and after NIR irradiation. **b** TEM image of Au@SiNRs after 5 irradiation cycles.

Both UV absorption curve and morphology of Au@SiNRs change little after the NIR irradiation, verifying their strong photothermal stability.

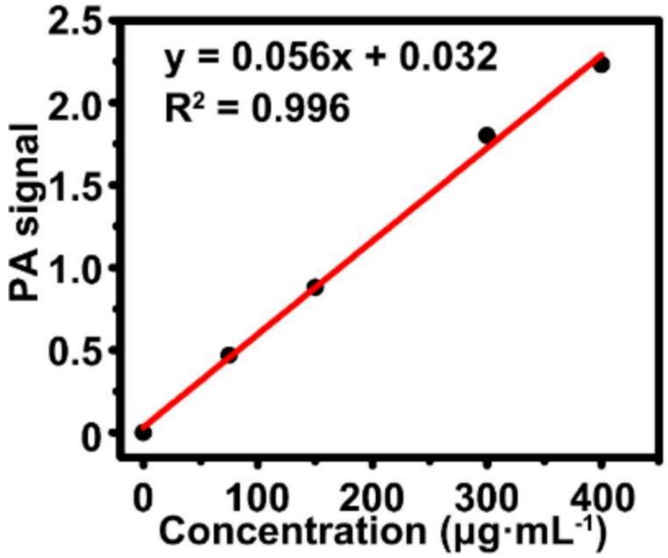

Fig. S9 Plot and linear fit of concentration of Au@SiNRs versus their PA signal intensity.

The quantitative analysis of photoacoustic signal shows that there is a remarkable positive correlation between the PA signal intensity and concentration of Au@SiNRs (R^2=0.996).

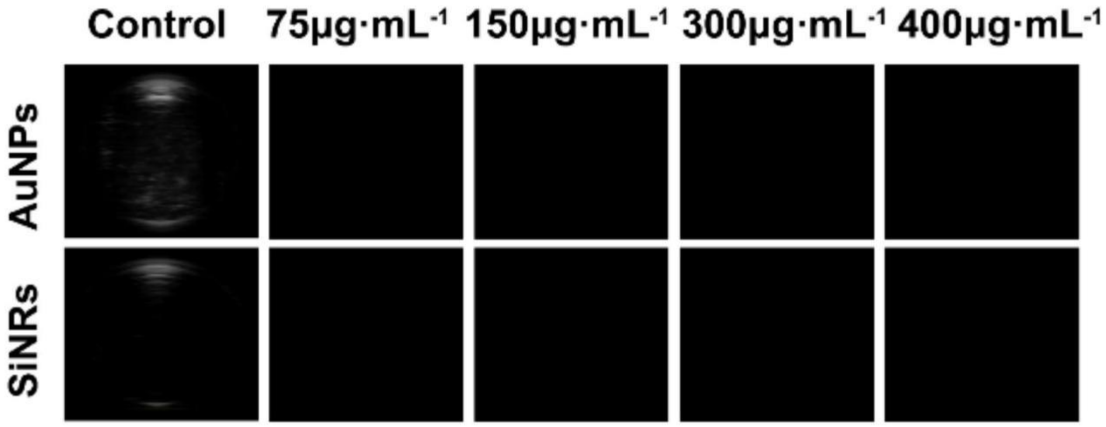

Fig. S10 PA effects of AuNPs and SiNRs with different concentrations.

It is obvious that neither AuNPs nor SiNRs are able to generate PA signal, even at relatively high concentrations (e.g., 300 or 400 μg·mL^{-1}).

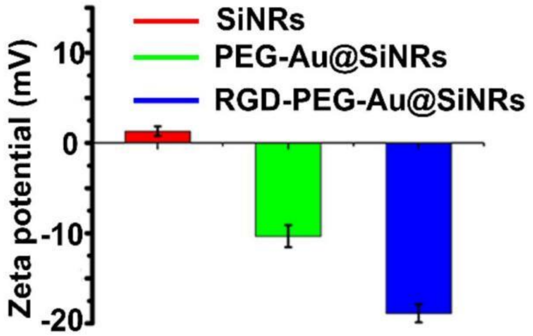

Fig. S11 Zeta potential of SiNRs, PEG-Au@SiNRs and RGD-PEG-Au@SiNRs.

Pristine SiNRs have negligible positive charge (1.3 ± 0.5 mV), while PEG-Au@SiNRs and RGD-PEG-Au@SiNRs are negative charge (-10.3 ± 1.2 and -18.9 ± 1.0 mV, respectively).

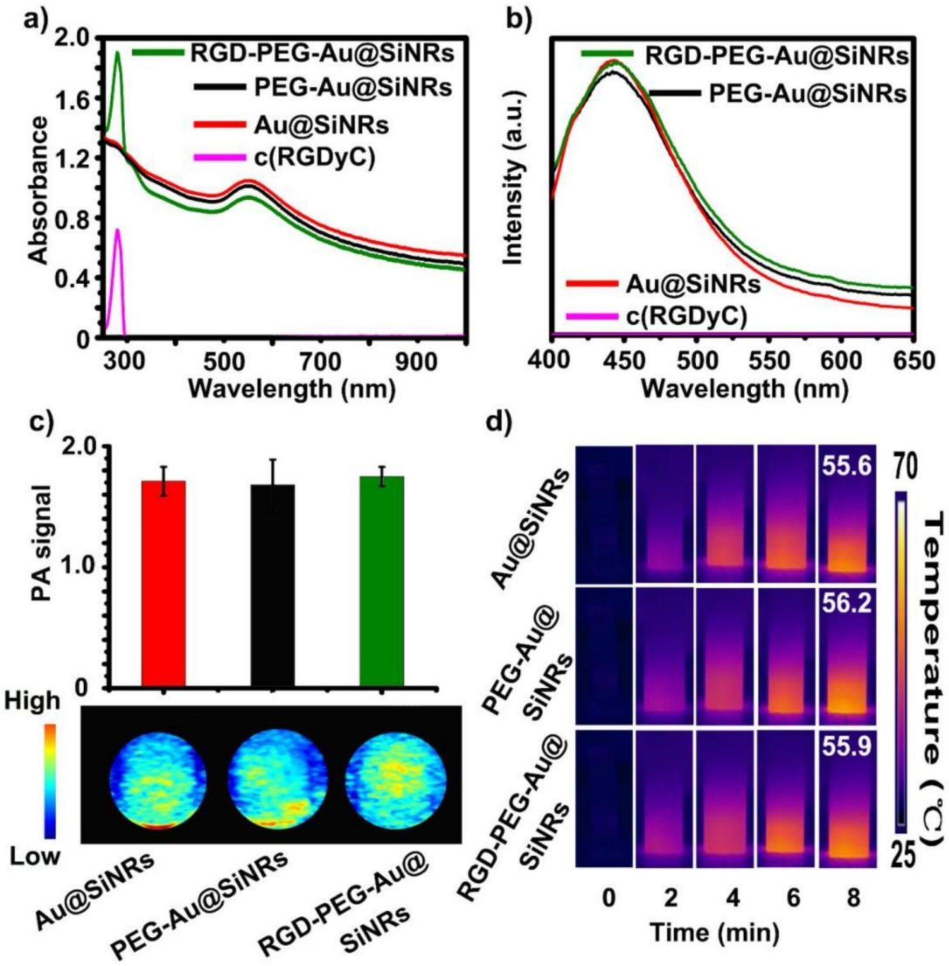

Fig. S12 Effect of surface modification on Au@SiNRs. a UV Absorption and **b** fluorescence spectra of Au@SiNRs, RGD-PEG-Au@SiNRs, PEG-Au@SiNRs (same concentrations according to Au@SiNRs) and free c(RGDyC) peptides. **c** PA and **d** heat signal generated by Au@SiNRs, RGD-PEG-Au@SiNRs and PEG-Au@SiNRs with

same concentrations according to Au@SiNRs.

According to Fig. S12a, b, surface modification of both PEG and c(RGDyC) peptides has no effect on the position and intensity of the characteristic UV absorbance and PL peak of Au@SiNRs. In addition, surface modification does not apparently affect the c) PA signal intensity and d) heat signal of Au@SiNRs.

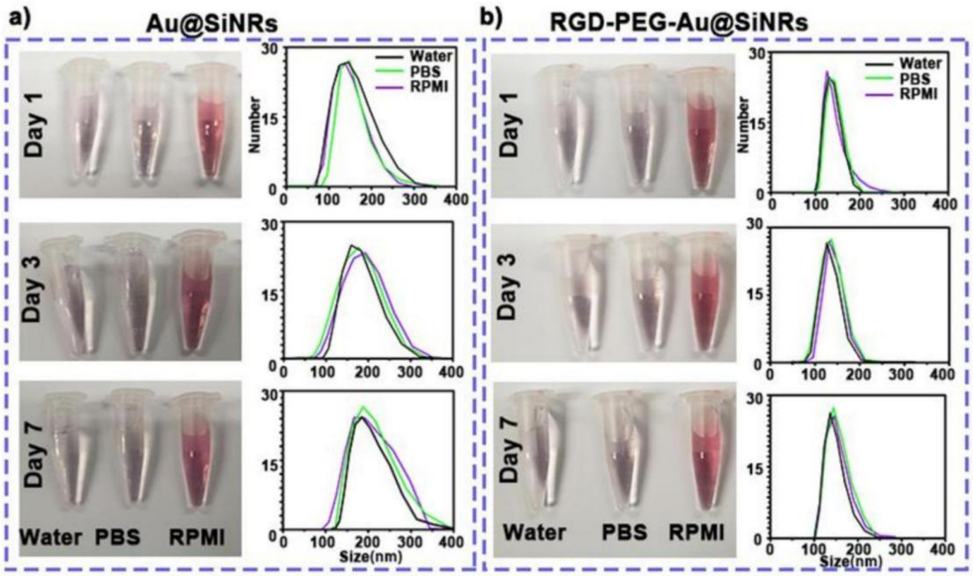

Fig. S13 Photos and DLS spectra of **a** Au@SiNRs and **b** RGD-PEG-Au@SiNRs (1mg/ml) in water, PBS (pH 7.4) and RPMI 1640 culture medium during 7-day storage.

In brief, it can be found the Au@SiNRs and RGD-PEG-Au@SiNRs have a good dispersion in water, PBS and culture medium during 7-day storage. Notably, the DLS spectra demonstrate that RGD-PEG-Au@SiNRs have narrower size distribution than Au@SiNRs.

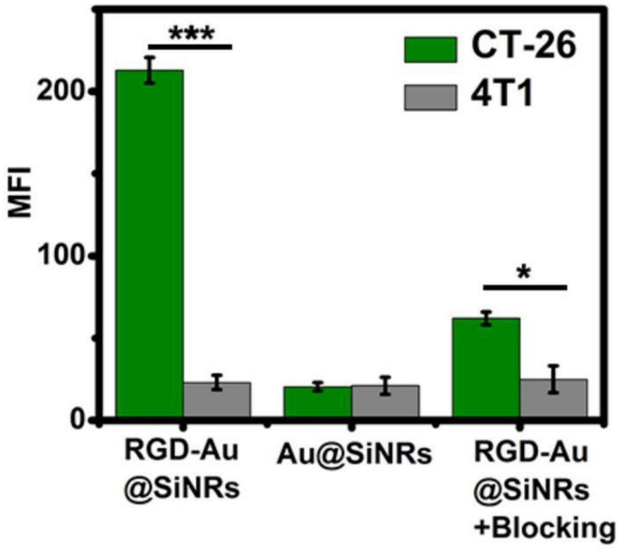

Fig. S14 The mean fluorescence intensity (MFI) in Fig. 3b was calculated by using

Leica LAS AF Lite software. Asterisk (*) indicates $p < 0.05$; (***) means $p < 0.001$.

The value of MFI in Fig.S14 shows that the RGD-Au@SiNRs-treated CT-26 cells exhibit stronger fluorescence compared with other groups.

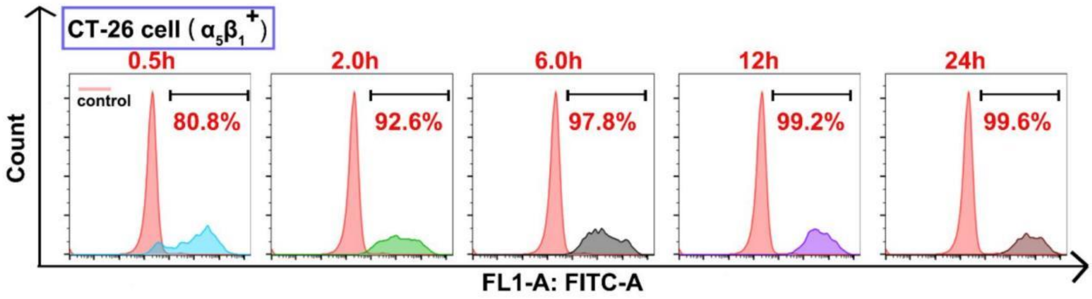

Fig. S15 Quantitative analysis of time-dependent uptake of RGD-Au@SiNRs by CT-26 cells through flow cytometry.

Flow cytometry is utilized to quantify the rate ofRGD-Au@SiNRs cellular association by analyzing the whole fluorescent intensity of 20 000 cells. As shown in Fig. S15, it can be found that after incubation for only 0.5 h, more than 80% cells have gained a strong fluorescence. Combined with the results shown in Fig. 3b, it can be speculated that the cellar uptake of RGD-Au@SiNRs by CT-26 cells should be integrin receptor mediated endocytosis, as reported in the other system of RGD-modified agents [1, 2].

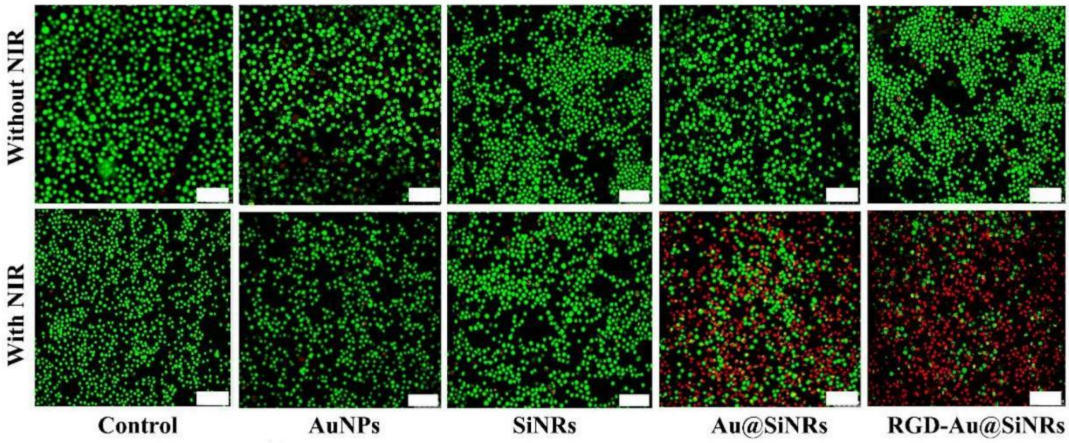

Fig. S16 Live/dead staining of CT-26 cells treated with PBS, AuNPs (7 μg mL^{-1}), SiNRs, Au@SiNRs and RGD-Au@SiNRs (200 μg mL^{-1}) for 4 h with or without 808 nm laser irradiation for 5 min at a power density of 0.8 W cm^{-2}.

AuNPs, SiNRs, Au@SiNRs and RGD-Au@SiNRs have no effect on the viability of CT-26 cells at the tested concentrations. In contrast, both Au@SiNRs and RGD-Au@SiNRs can lead to obvious photothermal ablation of cancer cells. In addition, the efficacy of RGD-Au@SiNRs is better dueto the targeting ability of c(RGDyC) peptides.

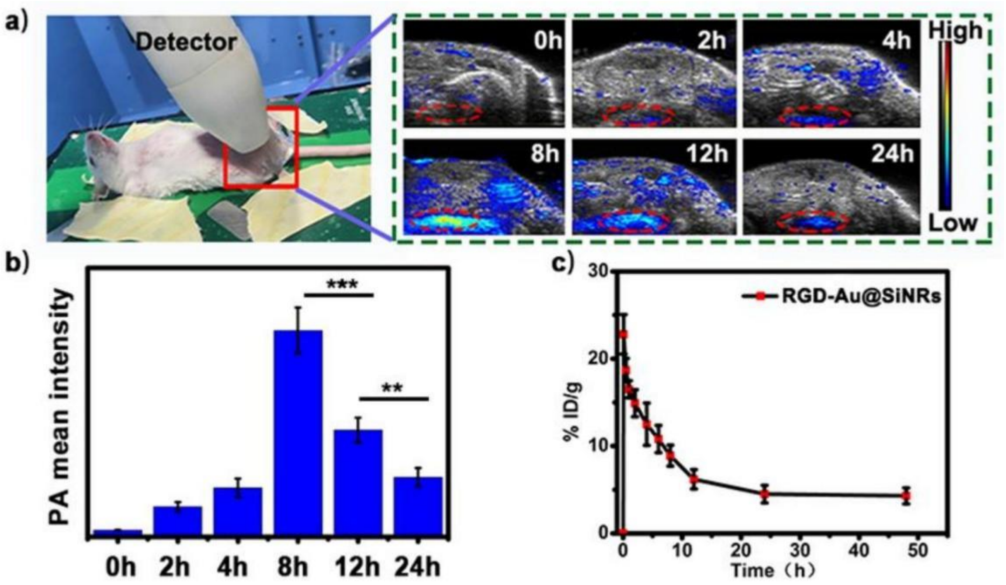

Fig. S17 PA signal of the bladders of mice and blood circulation of RGD-Au@SiNPs. **a** The bladder of mouse was detected by PA imaging system during 24 h and **b** the corresponding PA signal intensity. **c** The blood circulation spectrum of mice after intravenously (i.v.) injected with RGD-Au@SiNRs. Asterisk (**) indicates $p < 0.01$; (***) means $p < 0.001$.

As shown in Fig. S17a and S17b, the PA signal of bladder regions reached to the highest level after the injection with RGD-Au@SiNRs for 8 h, then gradually decreased. According to the curve line in Fig. S17c, the blood circulation half-life of intravenously injected RGD-Au@SiRNs was calculated to be ~ 4.0 hours.

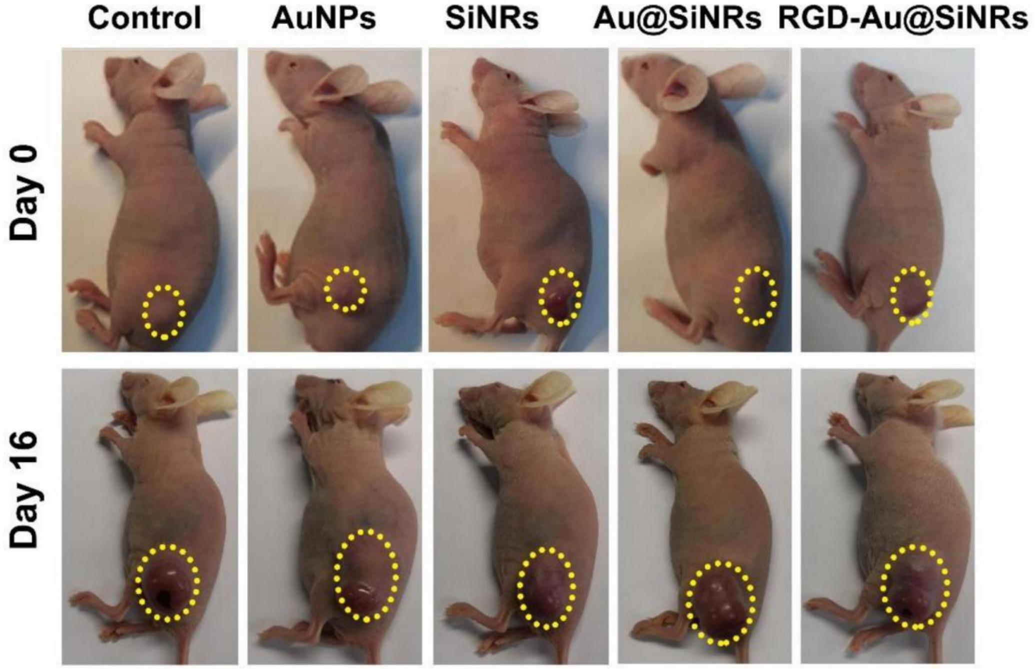

Fig. S18 Photos of representative mice treated with different agents without NIR irradiation.

Without NIR irradiation, none of PBS, AuNPs, SiNRs, Au@SiNRs and RGD-Au@SiNRs have effect on the growth of tumors.

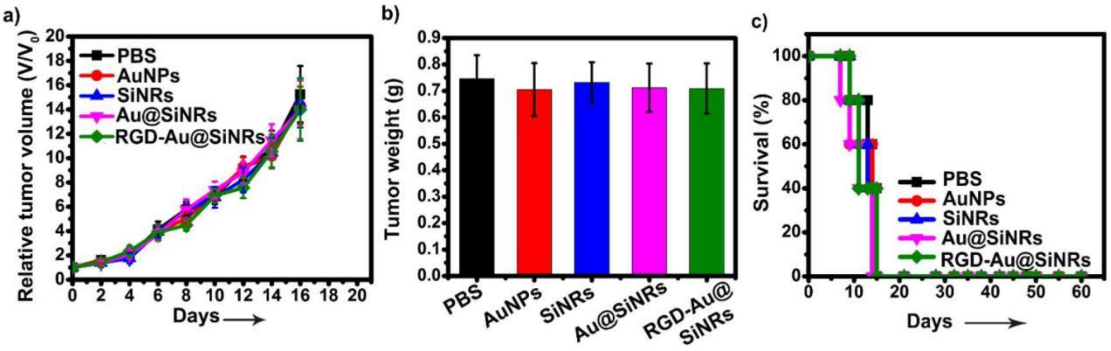

Fig. S19 a Relative tumor volume, **b** tumor weight, and **c** relative survival rate curves of mice from different groups without NIR treatment.

In the absence of NIR irradiation, AuNPs, SiNRs, Au@SiNRs and RGD-Au@SiNRs are unable to suppress the growth of tumors. After 16 days of treatment with PBS, AuNPs, SiNRs, Au@SiNRs or RGD- Au@SiNRs, the tumors increased in size by factors of 15.3, 14.3, 14.5, 14.0 and 14.0, respectively (Fig. S14 (a)); while the tumor weights increased to 0.75, 0.71, 0.73, 0.71 and 0.71 g, respectively (Fig. S14 (b)). There were no obvious differences in tumor volume or weight between these groups. Consequently, the survival time of tumor-bearing mice treated with Au@SiNRs or RGD-Au@SiNRs were not prolonged in comparison with PBS (Fig. S14 (c)).

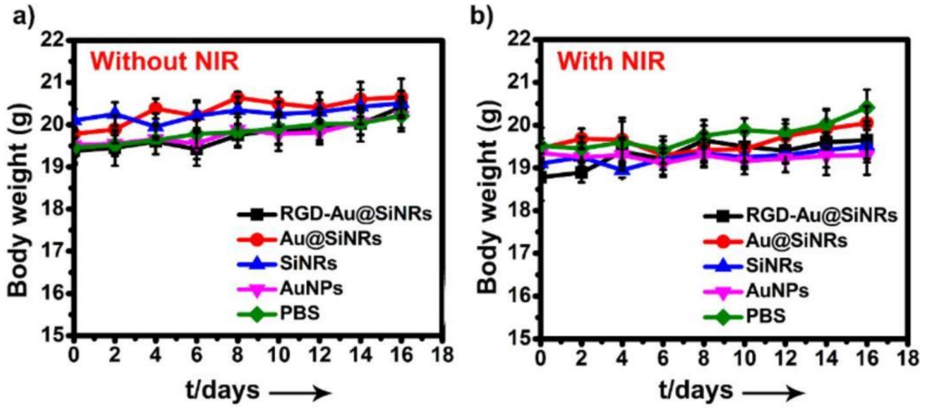

Fig. S20 Body weight of mice from different groups for 16 days after various treatments **a** without or **b** with NIR irradiation.

Administration of PBS (control), AuNPs (0.62 mg kg^{-1}), SiNRs, Au@SiNRs and RGD-Au@SiNRs (20 mg kg^{-1}) with or without NIR irradiation has almost no effect on the body weight of treated healthy mice.

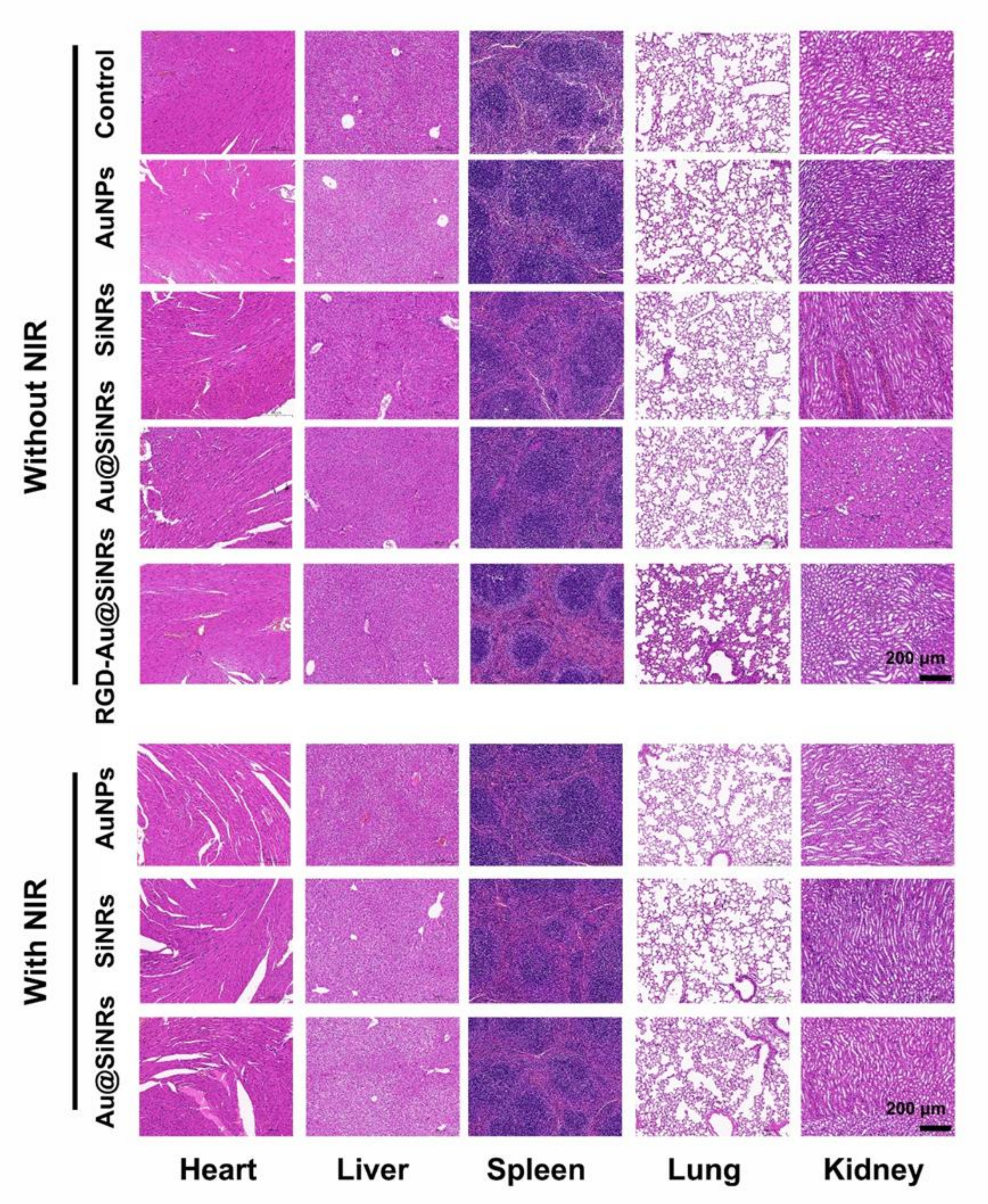

Fig. S21 H&E staining of various organ tissues harvested from mice at the end of treatment.

Mice were intravenously administered with PBS (control), AuNPs (0.62 mg·kg^{-1}), SiNRs, Au@SiNRs and RGD-Au@SiNRs (20 mg·kg^{-1}). After 24 h, tumor region was irradiated or not with an 808 nm laser for 10 min at a power density of 0.8 W·cm^{-2}. After 16 days of treatment, the major organs (heart, liver, spleen, lung and kidney) were harvested from different groups of mice for H&E staining.

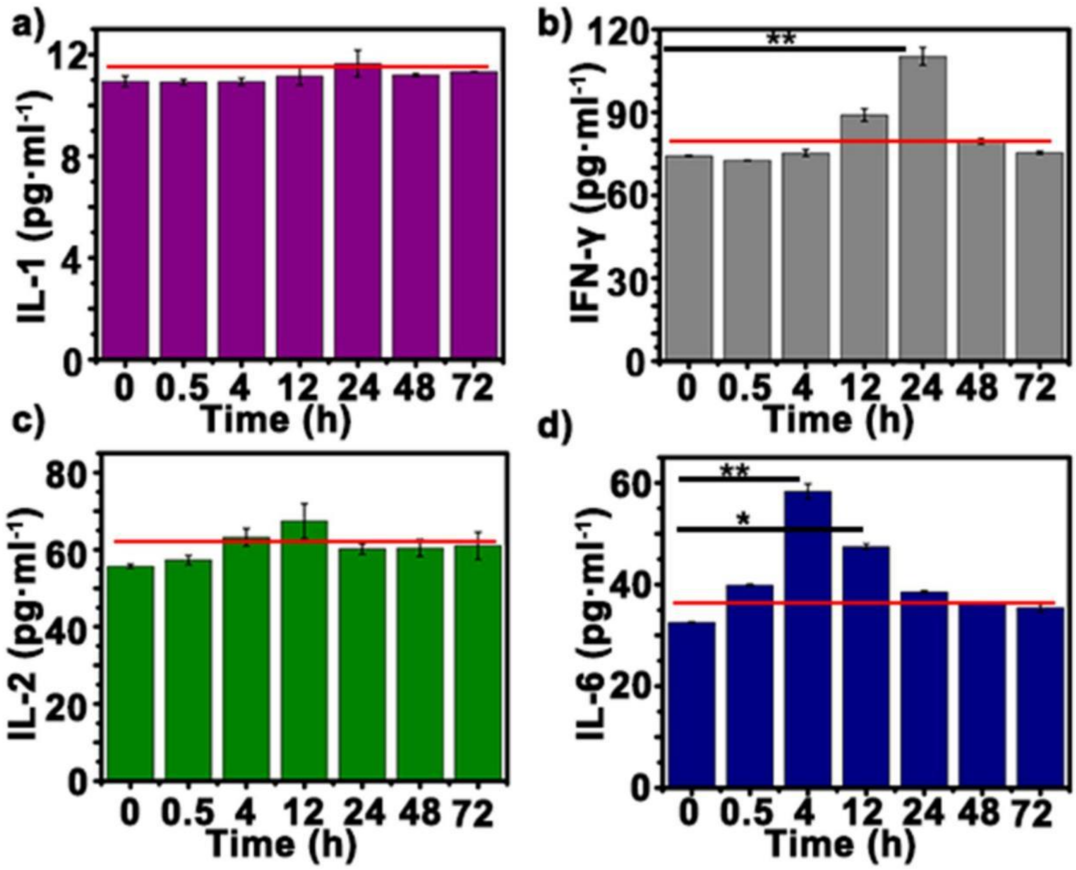

Fig. S22 Effect of RGD-Au@SiNRs (20mg kg^{-1}) on the blood levels of **a** IL-1, **b** IFN-γ, **c** IL-2 and **d** IL-6. Asterisk (*) indicates $p < 0.05$; (**) means $p < 0.01$.

The levels of several inflammatory cytokines released to the serum in treated mice were elevated after i.v. injection of RGD-Au@SiNRs (20mg kg^{-1}). There was slight change in IL-1 and IL-2 during 72 h. IL-6 reached to maximum at 4 h after treatment and the level was decreased gradually in a time-dependent manner to 24 h; while, IFN-γ reached to maximum at 24 h after treatment and the level was decreased to normal at 48 h.

References:

[S1] C. Zhang, M. Jugold, E. C. Woenne, T. Lammers, B. Morgenstern, M. M. Mueller, H. Zentgraf, M. Bock, and M. Eisenhut, W. Semmler, F. Kiessling, Specific Targeting of Tumor Angiogenesis by RGD-Conjugated Ultrasmall Superparamagnetic Iron Oxide Particles Using a Clinical 1.5-T Magnetic Resonance Scanner. Cancer Res. **67**(4), 1555-1562 (2007).https://cancerres.aacrjournals.org/content/67/4/1555.

[S2] M. H. Lee, J. Y. Kim, J. H. Han, S. Bhuniya, J. L. Sessler, C. Kang, J. S. Kim, Direct Fluorescence Monitoring of the Delivery and Cellular Uptake of a Cancer-Targeted RGD Peptide-Appended Naphthalimide Theragnostic Prodrug. J. Am. Chem. Soc. **134**(30), 12668-12674 (2012).https://doi.org/10.1021/ja303998y
